# The Red Blood Cell–Liver Axis: A New Framework for Iron Homeostasis and Liver Biology

**DOI:** 10.3390/biology15191773

**Published:** 2026-10-06

**Authors:** Sebastian Mueller

**Affiliations:** Center for Alcohol Research, Heidelberg University Hospital, 69120 Heidelberg, Germany; sebastian.mueller@urz.uni-heidelberg.de

**Keywords:** bile formation, erythrocyte turnover, heme metabolism, hemolysis, hepcidin, iron homeostasis, liver evolution, liver physiology, red blood cell–liver axis, redox signaling

## Abstract

The liver is traditionally viewed as an organ of metabolism, bile formation, detoxification, and iron storage. Evidence shows that hepatic red blood cell (RBC) handling extends beyond macrophages to include uptake by hepatocytes and liver sinusoidal endothelial cells, forming a multicellular clearance network. This article introduces the RBC–liver axis, through which the liver senses, processes, and responds to RBC-derived material. Daily RBC turnover generates the dominant flux of heme and iron, recycling 25 mg of iron per day, while hepatocytes couple heme-responsive NRF2 signaling to MRP2-mediated bilirubin excretion. Hepcidin emerges as an integrated output of RBC-derived heme and iron flux, inflammation, oxygen tension, redox status, and erythropoietic demand. Evidence supports a biphasic response in which moderate RBC-derived heme and iron flux stimulates hepcidin, whereas excessive hemolysis may dysregulate this response through ERFE-dependent and ERFE-independent mechanisms. Mechanistically, this model integrates BMP6–SMAD signaling with HO-1, KEAP1–NRF2, BACH1, and redox-sensitive H_2_O_2_–STAT3 signaling amplified by hypoxia. Alcohol-associated liver disease illustrates its clinical relevance by linking enhanced erythrocyte turnover to iron accumulation and mortality. Thus, the liver emerges as a central processor of RBC biology, whose metabolic, protein-producing, lipid-handling, detoxifying, and biliary functions may reflect an evolutionarily conserved blood-processing program.

## Simple Summary

The liver is best known for metabolism, detoxification, bile production, and iron storage. However, it also continuously processes material from aging or damaged red blood cells. These cells are removed not only by macrophages in the spleen and liver but also by liver endothelial cells and hepatocytes. Together, these cells handle hemoglobin, heme, iron, membrane lipids, cholesterol, and bilirubin precursors. The red blood cell–liver axis describes how the liver senses this continuous flow and adapts its metabolism to it. Moderate red blood cell turnover may stimulate hepcidin, the hormone that controls iron distribution. In contrast, severe hemolysis or heme overload may suppress or disrupt this response through erythropoietic signals, heme degradation, oxidative stress, and changes in oxygen and redox signaling. This axis is clinically important in alcohol-associated liver disease, where red blood cell damage, hemolysis, iron accumulation, and poor outcomes are closely linked. The concept places the liver at the center of red blood cell processing and suggests that many of its classical functions—including detoxification, protein and lipid handling, antioxidant defense, and bile formation—may have developed partly to process blood-cell components and safely eliminate their potentially toxic breakdown products.

## 1. The Liver Beyond Metabolism: An Overlooked Red Blood-Cell Interface

The liver is traditionally viewed as the central metabolic organ of the body, coordinating carbohydrate, lipid, protein, and bile acid metabolism while maintaining systemic energy homeostasis. However, another fundamental function may have remained largely overlooked: its continuous interaction with red blood cells and their degradation products. Every day, under normal conditions, approximately one percent of the circulating erythrocyte mass is replaced, generating a substantial flux of hemoglobin, heme, iron, membrane lipids, and bilirubin precursors that must be processed safely and efficiently [1,2]. Given their abundance and continuous turnover, erythrocytes may constitute one of the largest uniform endogenous cellular and self-antigenic loads subjected to daily phagocytic clearance and recycling, thereby linking the RBC–liver axis to hepatic immune tolerance. While individual components of this pathway have long been recognized, including bilirubin excretion, iron handling, and macrophage-mediated erythrophagocytosis, they have rarely been considered as parts of a unified biological system. Recent studies in alcohol-associated liver disease have revealed a previously underrecognized erythrocyte-driven injury pathway in which increased erythrocyte fragility, eryptosis, and hemolysis converge to link disturbed red blood cell homeostasis with overall mortality, hepatic injury, fibrosis, and iron accumulation [3]. In parallel, experimental evidence indicates that, in addition to macrophages, liver sinusoidal endothelial cells (LSEC) and hepatocytes can directly ingest them [4,5].

These observations raise the possibility that the liver is not merely a downstream recipient of erythrocyte-derived metabolites but represents a central cellular interface that continuously monitors, processes, and responds to red blood cell turnover. Such a perspective may help integrate a wide range of seemingly unrelated phenomena into a common conceptual framework that can be described as the red blood cell–liver axis.

## 2. Enhanced Erythrocyte Turnover Determines Mortality: Clinical Evidence from a 15-Year Prospective Cohort of Heavy Drinkers

**Figure 1:**
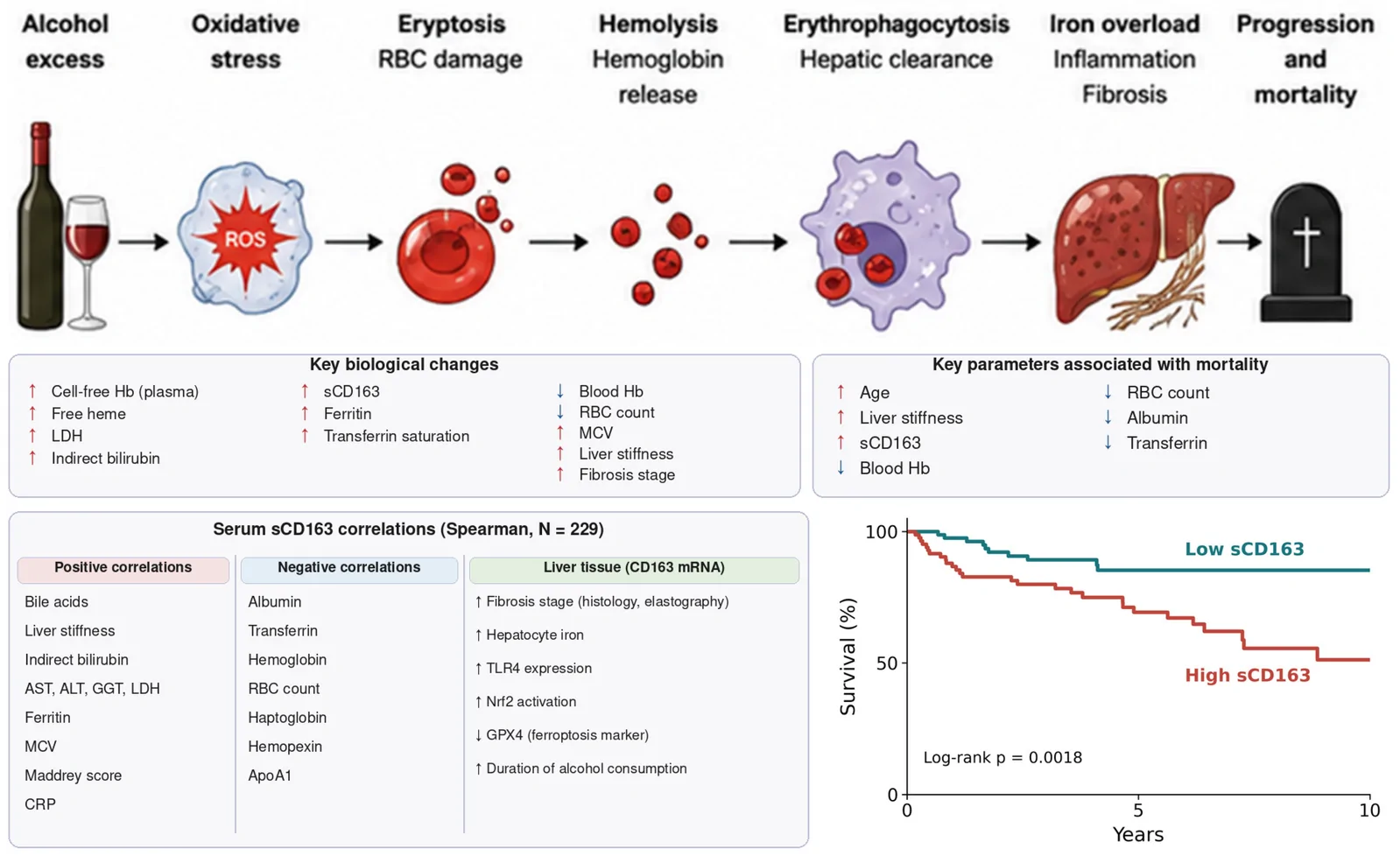
Erythrocyte turnover, hemolysis, macrophage activation, and long-term mortality in alcohol-associated liver disease. Proposed relationship between erythrocyte turnover, hemolysis, iron metabolism, and mortality in alcohol-associated liver disease (ALD). Chronic alcohol exposure induces oxidative stress, eryptosis, low-grade hemolysis, hepatic erythrophagocytosis, and continuous delivery of heme-derived iron to the liver [3]. This may promote iron accumulation, oxidative stress, inflammation, fibrosis progression, and adverse outcome. Cell-free hemoglobin denotes hemoglobin released into plasma and is distinct from the hemoglobin concentration measured in the blood count. In a prospective cohort of heavy drinkers followed for up to 15 years, higher age, liver stiffness, and sCD163 and lower blood hemoglobin, RBC count, albumin, and transferrin were associated with mortality in univariable analyses. High baseline sCD163 was linked to reduced long-term survival in the Kaplan–Meier analysis (*n* = 173; median split at 1407.21 ng/mL; log-rank *p* = 0.0018). The curves are displayed through 10 years, supporting the concept that disturbed erythrocyte turnover is biologically relevant in ALD. Abbreviations: ALD, alcohol-associated liver disease; ALT, alanine aminotransferase; ApoA1, apolipoprotein A1; AST, aspartate aminotransferase; CRP, C-reactive protein; GGT, gamma-glutamyltransferase; GPX4, glutathione peroxidase 4; Hb, hemoglobin; LDH, lactate dehydrogenase; MCV, mean corpuscular volume; Nrf2, nuclear factor erythroid 2-related factor 2; RBC, red blood cell; ROS, reactive oxygen species; sCD163, soluble CD163; TLR4, Toll-like receptor 4. Serum sCD163 correlations and liver-tissue findings are adapted from the Appendix of reference [7]; the Kaplan–Meier curves were calculated from the cohort data.

One of the strongest clinical arguments for a biologically relevant red blood cell–liver axis comes from long-term outcome data in heavy drinkers. In a prospective Heidelberg cohort, 786 heavy drinkers were followed for up to 15 years. Of these, ca. 20% died during a mean observation period of approximately 4 years, corresponding to an approximately fourfold age-adjusted mortality risk compared with the general population (Figure 1) [6]. More details of the cohort are provided in the Appendix of [6]. The excess risk was particularly pronounced at younger ages. These findings demonstrate the profound premature mortality associated with heavy alcohol consumption and raise the question of which biological processes contribute to this outcome.

Figure 1 also provides key biological consequences and univariate and multivariate predictors of mortality from this cohort. As expected, liver stiffness showed the strongest univariate association with mortality, confirming the central role of advanced liver disease [6,8]. Unexpectedly, however, the erythrocyte compartment showed similarly prominent prognostic relevance [6,9]. Erythrocyte count displayed one of the strongest inverse associations with survival and outperformed hemoglobin, hematocrit, and several established prognostic laboratory parameters. Reduced erythrocyte counts were accompanied by increased total and indirect bilirubin, LDH, ferritin, and MCV, consistent with enhanced erythrocyte turnover. Indirect bilirubin and soluble CD163, reflecting activation of the macrophage hemoglobin–haptoglobin scavenging system [10], were among the laboratory parameters also most strongly associated with death. In multivariate Cox regression, erythrocyte count remained an independent predictor of mortality together with age, alkaline phosphatase, and total bilirubin, whereas liver stiffness remained part of the prognostic model but did not reach conventional statistical significance. Thus, the erythrocyte compartment provided prognostic information beyond conventional measures of hepatic injury and fibrosis.

Enlarged erythrocytes, reflected by an elevated mean corpuscular volume (MCV) and clinically termed macrocytosis, are a characteristic hallmark of alcohol-related disease and provide an accessible entry point into this erythrocyte-related phenotype. Increased MCV is classically observed in vitamin B12 or folate deficiency and during reticulocytosis in response to hemolysis, but it has also been recognized for decades as a characteristic laboratory feature of chronic alcohol consumption [11]. The classical Zieve syndrome, first described in 1958 and characterized by hemolytic anemia, jaundice, and transient hyperlipidemia in the setting of heavy alcohol use, represents a rare but instructive clinical manifestation of alcohol-associated hemolysis [12]. In the Heidelberg cohort, elevated MCV was not merely an isolated or nonspecific marker of alcohol exposure but identified a broader erythrocyte-turnover phenotype. Patients with high MCV showed increased sCD163, a higher prevalence of elevated sCD163 and ferritin, a higher anemia fraction, altered erythropoietic and iron-regulatory parameters, and the highest liver injury and mortality (Supplementary Table S1) [9]. Of note, folic acid was normal and B12 levels were even elevated in the high MCV group.

Further clinical and experimental observations support the interpretation that this laboratory phenotype reflects increased erythrocyte vulnerability and destruction. After centrifugation, serum from heavy drinkers was visibly red, representing the classical macroscopic sign of hemolysis; this finding largely disappeared after one week of alcohol detoxification [3]. In patients with severe alcohol-associated liver disease, erythrocytes were more fragile and exhibited approximately 2.5-fold greater hemolysis under standardized hemolytic stress conditions in vitro as compared to erythrocytes from healthy controls [3]. Experimental studies further demonstrated enhanced eryptosis, externalization of phosphatidylserine and rapid erythrophagocytosis following ethanol exposure [3,4]. Importantly, hemoglobin could be detected in the livers of both ethanol-exposed mice and heavy drinkers across macrophages, liver sinusoidal endothelial cells, and hepatocytes, supporting a multicellular hepatic network for the uptake and processing of RBC-derived material [3,4].

On a final note, alcohol may simultaneously impair compensatory erythropoiesis through direct toxicity to erythroid precursor cells and disruption of late erythroid differentiation [13,14,15,16]. As recently discussed on the basis of a chronic murine alcohol model, chronic alcohol exposure may suppress hematopoietic stem and early progenitor compartments while simultaneously driving compensatory but ineffective late erythroblast proliferation and maturation, thereby coupling increased erythrocyte destruction to insufficient production of functional mature RBCs [16].

Taken together, emerging human data identify accelerated RBC turnover and hepatic uptake, processing, and excretion of erythrocyte-derived material as an unexpected and clinically relevant component of alcohol-related disease that is strongly associated with mortality.

## 3. Evolutionary Origins of the Liver–Blood Axis

The close relationship between the liver and circulating blood is deeply rooted in vertebrate evolution and embryonic development. During mammalian embryogenesis, the fetal liver becomes the principal hematopoietic organ after the initial waves of blood formation in the yolk sac. Hematopoietic stem and progenitor cells colonize the developing liver, where they undergo extensive expansion and differentiation before definitive hematopoiesis shifts to the bone marrow [17,18]. The close association between hepatic tissues and blood-cell production is conserved across vertebrate development and evolution, although the anatomical organization of hematopoietic organs differs among species [19,20]. In many vertebrates, including fish and amphibians, blood formation and blood-cell turnover remain linked to hepatic or hepatic-associated tissues. In adult mammals, this ancestral relationship persists in hepatic erythrocyte clearance, heme degradation, iron recycling, bilirubin excretion, and hepcidin-mediated regulation of systemic iron homeostasis.

The evolutionary origins of iron regulation extend even further. Hepcidin-like peptides occur across diverse animal lineages and combine antimicrobial activity with iron-regulatory functions [21,22]. This supports the concept that iron sensing and host defense co-evolved before their integration into the highly specialized mammalian hepcidin–ferroportin system. During vertebrate evolution, these functions became increasingly coordinated within the liver. The modern liver may therefore retain elements of an ancient biological program linking hematopoiesis, erythrocyte turnover, heme metabolism, iron recycling, and antimicrobial defense.

This evolutionary framework helps explain why erythrocyte clearance, heme metabolism, iron homeostasis, and hepcidin regulation remain so tightly integrated within the liver. Rather than representing independent pathways that subsequently converged in the same organ, they may reflect different components of a common blood-processing program. Several functions now considered characteristic of the liver—including redox defense, plasma-protein production, lipid and cholesterol handling, detoxification, and biliary excretion—may have evolved in close association with the continuous requirement to process erythrocytes and safely eliminate their potentially toxic degradation products. The fetal role of the liver in hematopoiesis and its persistent adult functions in erythrocyte clearance, heme degradation, iron recycling, and bilirubin excretion support the concept that this blood-processing program preceded or accompanied the emergence of its broader metabolic functions. Remarkably, as early as 1858, Frerichs connected the red constituents of blood and hematin with bile pigment and described how pigment particles and other products arising from splenic disease enter the portal circulation and affect the liver, thereby anticipating a systemic relationship between erythrocyte breakdown, the spleen, and hepatic processing [23].

## 4. The Daily Erythrocyte Flux: A Familiar Number with Underestimated Consequences

**Figure 2:**
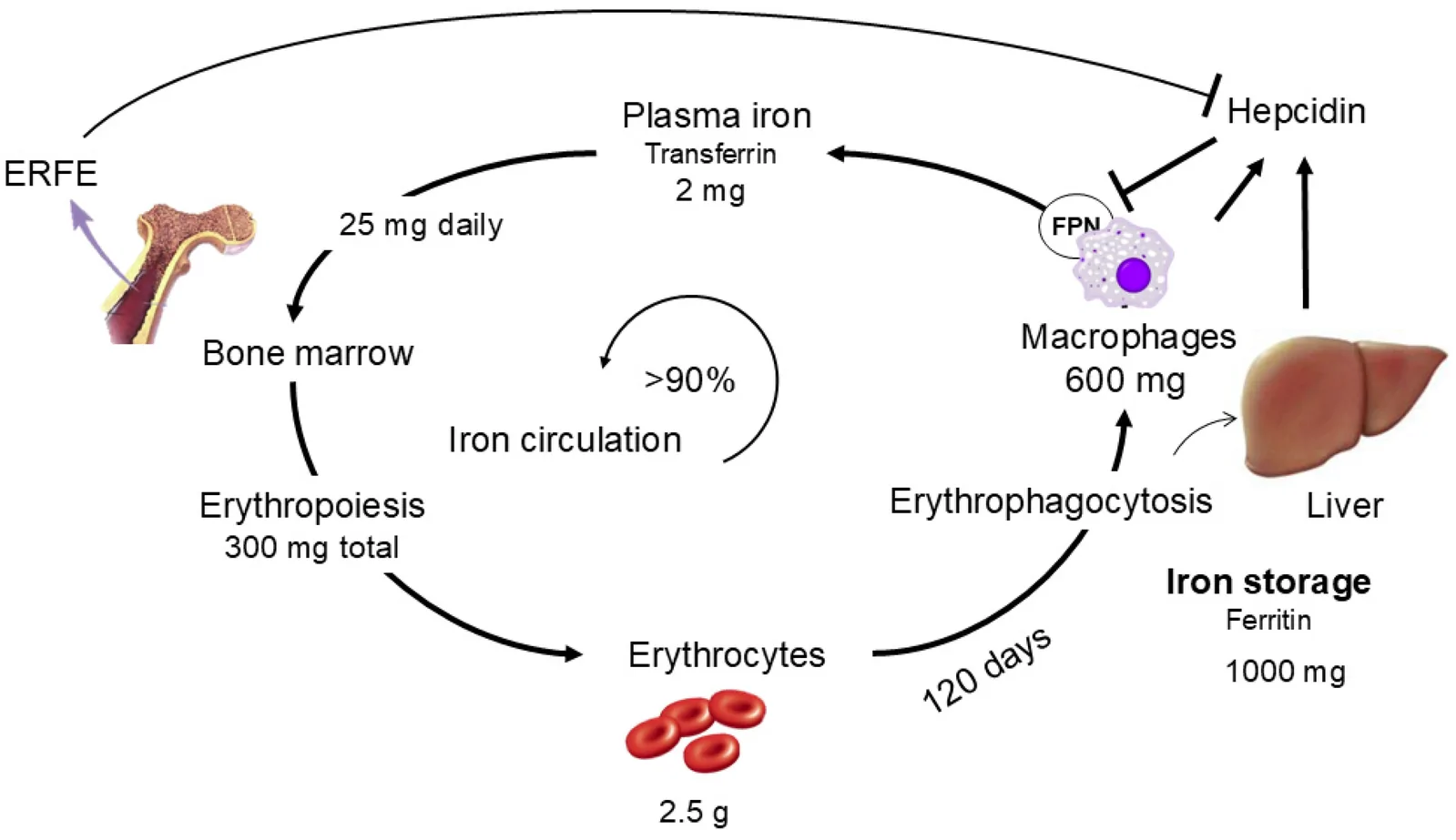
Classical depiction of physiological erythrocyte turnover, systemic iron recycling and hepcidin regulation. Systemic iron homeostasis is dominated by the continuous turnover of erythrocytes, which recycles approximately 20–25 mg of iron per day, accounting for more than 90% of the daily iron flux. For decades, macrophages were considered the principal cells responsible for erythrocyte clearance. Iron released from senescent erythrocytes after approximately 120 days of circulation is recovered by macrophages through erythrophagocytosis and exported via ferroportin (FPN) into the plasma transferrin pool. The recycled iron is subsequently delivered to the bone marrow to sustain erythropoiesis, ultimately generating an erythrocyte iron mass of approximately 2.5 g. Excess iron is stored predominantly in the liver as ferritin (approximately 1000 mg under physiological conditions). Hepcidin, mainly secreted by hepatocytes, acts as the master regulator of systemic iron homeostasis by inducing internalization and degradation of ferroportin on macrophages, thereby restricting iron release into the circulation. Conversely, increased erythropoietic activity stimulates erythroferrone (ERFE) production in the bone marrow, which suppresses hepatic hepcidin expression and thereby promotes iron mobilization to support erythropoiesis. This feedback system couples erythrocyte turnover, iron recycling, hepatic iron storage, and erythropoietic demand into a tightly regulated physiological cycle. Abbreviations: ERFE, erythroferrone; FPN, ferroportin. Modified from Ref. [24].

Red blood cell turnover is one of the most fundamental physiological processes in humans, yet its quantitative magnitude and metabolic consequences are often underestimated (Figure 2). With an average lifespan of approximately 120 days, nearly 1% of the circulating erythrocyte mass is replaced every day. Assuming an adult blood volume of approximately 5 L and an average erythrocyte count of approximately 5 × 10^12^ cells per liter, the total circulating erythrocyte mass is approximately 2.5 × 10^13^ cells. Dividing this number by an average erythrocyte lifespan of approximately 120 days yields a daily turnover of approximately 2 × 10^11^ erythrocytes. Based on an average erythrocyte volume of approximately 90–100 fL, this corresponds to approximately 18–20 mL of erythrocyte volume per day [1,2]. Viewed in terms of organ-scale mass, a liver weighing approximately 1.5 kg continuously interfaces with a circulating packed erythrocyte mass of roughly 2–2.5 kg, about 1% of which is renewed each day. Given their abundance and continuous turnover, erythrocytes may constitute one of the largest—possibly the largest—uniform endogenous cellular and self-antigenic loads subjected to daily phagocytic clearance and recycling, thereby linking the RBC–liver axis to hepatic immune tolerance.

Although this process is continuous throughout life, it rarely attracts attention because under physiological conditions it remains remarkably efficient and largely invisible. Moreover, RBC turnover is not readily accessible in routine clinical practice; direct quantification requires specialized red-cell survival techniques, such as chromium-51 or biotin labeling, whereas most clinical studies rely on indirect surrogate markers of hemolysis and erythrocyte clearance [25]. The daily disposal of aging erythrocytes generates a substantial flux of biologically active molecules, including hemoglobin, heme, iron, membrane phospholipids, cholesterol, amino acids, carbon monoxide, biliverdin, and bilirubin precursors [26]. Part of this continuous flux may occur through RBC-derived extracellular vesicles, which allow erythrocytes to remove hemoglobin, oxidized proteins, and membrane components during aging before the cells themselves are cleared [27]. Traditionally, these products have been studied individually within separate disciplines such as iron metabolism, bilirubin metabolism, or oxidative stress biology. However, they all originate from a common source: the continuous turnover of circulating erythrocytes.

For decades, and as shown in the classical depiction of RBC recycling (Figure 2), macrophages were considered the principal cells responsible for erythrocyte clearance [28]. More recent observations suggest a more complex picture. In addition to Kupffer cells, both liver sinusoidal endothelial cells and even hepatocytes can mediate the phosphatidylserine-dependent recognition, sequestration and subsequent uptake and processing [4,5]. However, the relative quantitative contributions of Kupffer cells, hepatocytes and LSECs remain unresolved, as the available histological studies do not yet permit a direct comparison of total clearance capacity, uptake kinetics, or downstream intracellular processing. Thus, erythrocyte turnover is not simply a hematological phenomenon but involves an extensive hepatic clearance network operating at the interface between the circulation and the liver. Direct ingestion of RBCs by hepatocytes is of particular importance, since hepatocytes are the principal hepatic cells responsible for bilirubin conjugation and its canalicular export into bile via the export pump MRP2 [29,30,31]. This represents the principal physiological route of bilirubin disposal, although alternative routes may become relevant under pathological conditions such as severe cholestasis or advanced liver disease.

From a systems perspective, the liver is therefore continuously exposed to erythrocyte-derived material. Under physiological conditions, this exposure is tightly controlled through coordinated processes of eryptosis, erythrophagocytosis/efferocytosis, heme degradation, bilirubin formation, and iron recycling. Yet even modest disturbances in erythrocyte survival may substantially increase the amount of heme, iron, and membrane-derived metabolites delivered to hepatic cells. Consequently, pathological conditions associated with enhanced erythrocyte turnover, including alcohol exposure, infections, oxidative stress, hemolytic disorders, ineffective erythropoiesis, and advanced liver disease, may profoundly influence liver function even in the absence of major changes in dietary iron intake [3].

Viewed from this perspective, erythrocyte turnover is not a peripheral process occurring independently of liver biology. Rather, it represents a major physiological flux continuously shaping hepatic metabolism, iron homeostasis, oxidative stress responses, and biliary function. Understanding this daily erythrocyte-driven metabolic burden provides the conceptual basis for a broader view of the liver as a central organ of erythrocyte processing rather than merely a recipient of erythrocyte degradation products.

## 5. Systemic and Cellular Iron Homeostasis

Hepatic iron accumulation is a common pathological feature of systemic iron-overload disorders [32] and many chronic liver diseases, including alcohol-associated liver disease (ALD), the most common liver disease worldwide [24] (Supplementary Figure S1). As illustrated in Supplementary Figure S1, pathological iron deposits become visible by Prussian blue staining and show distinct distribution patterns. Whereas iron in hereditary hemochromatosis is predominantly found in periportal hepatocytes, quantitative histological analysis in patients with alcohol-associated liver disease showed that hepatic iron deposits were distributed approximately equally between macrophages and hepatocytes [24].

Excess intracellular iron is biologically relevant because ferrous iron catalyzes the Fenton reaction, generating highly reactive hydroxyl radicals from hydrogen peroxide. These radicals promote oxidative stress, lipid peroxidation, mitochondrial dysfunction, DNA damage, and ultimately fibrosis and carcinogenesis [33]. Iron overload is therefore not merely a marker of liver disease but an active contributor to disease progression.

To minimize this toxicity, iron metabolism is regulated at both the cellular and systemic levels [34,35]. Individual cells autonomously adjust their intracellular iron content through the iron regulatory proteins IRP1 and IRP2, which bind iron-responsive elements (IREs) within target mRNAs [36,37,38]. Under iron deficiency, IRPs increase transferrin-receptor expression while suppressing ferritin synthesis, thereby promoting iron uptake and limiting storage. Conversely, when intracellular iron is abundant, IRPs become inactive, allowing ferritin synthesis while reducing further iron uptake. This IRP–IRE system enables every cell to maintain its own intracellular iron balance. Accordingly, ferritin induction should not be interpreted solely as an acute-phase response but also as a cell-autonomous consequence of intracellular iron loading in macrophages, hepatocytes, and other cell types, particularly when hepcidin-mediated ferroportin degradation restricts cellular iron export.

At the systemic level, most iron entering the circulating transferrin pool under physiological conditions is derived from the daily recycling of senescent erythrocytes rather than from intestinal absorption. Macrophages recover approximately 20–25 mg of iron per day, compared with only 1–2 mg absorbed from the intestine, and release it through ferroportin for transferrin-mediated delivery predominantly to the erythroid marrow [39]. This quantitatively dominant erythrocyte-derived iron flux provides the established physiological background for the proposed RBC–liver axis and links cellular iron handling to hepatic control of systemic iron distribution.

Systemic iron homeostasis is coordinated by the hepatic hormone hepcidin (see also Figure 2), whose central regulatory role was established by the severe iron-overload phenotype observed in mice lacking hepatic hepcidin expression [40]. Hepcidin binds to the sole known cellular iron exporter ferroportin, inducing its internalization and degradation [41]. Consequently, intestinal iron absorption and iron release from macrophages decrease, while iron is retained within hepatic and other cellular stores. Hepcidin synthesis in hepatocytes is primarily regulated through the BMP6–HFE–HJV–SMAD pathway, with iron-responsive BMP6 produced predominantly by liver sinusoidal endothelial cells [42], while inflammatory cytokines additionally stimulate hepcidin through STAT3 signaling [43,44]. Together, these pathways maintain systemic iron homeostasis under physiological conditions. Additional cellular signals, including sustained H_2_O_2_ and low oxygen tension, stimulate or amplify hepcidin through redox-sensitive STAT3 signaling [45,46], but do not replace the underlying BMP–SMAD-dependent framework required for the hepcidin response.

The major pathways regulating hepcidin are summarized in Supplementary Figure S2. In addition to the canonical BMP6–SMAD, inflammatory JAK–STAT3, and erythroferrone pathways, sustained submicromolar H_2_O_2_ induces hepatocellular hepcidin through STAT3, and low oxygen tension further amplifies this response through NOX4 and STAT3 [45,46]. These established oxygen–redox mechanisms provide the basis for the later discussion of how heme-derived CO and heme-dependent NO may connect erythrocyte turnover with mitochondrial respiration, redox signaling, and hepcidin regulation. They should be understood as complementary cellular signals acting within the essential BMP–SMAD-dependent framework of hepcidin regulation, rather than as an alternative pathway that can independently maintain the hepcidin response.

Although the importance of daily RBC turnover is well recognized, iron homeostasis has traditionally been viewed primarily in terms of intestinal iron absorption, macrophage-mediated recycling, and iron utilization for the de novo synthesis of iron-containing proteins, particularly hemoglobin in the bone marrow. The concept of the RBC–liver axis broadens this perspective by placing the liver’s role in RBC clearance and degradation, bilirubin excretion, and iron handling within a unified physiological framework.

## 6. Oxygen–Redox Coupling in Hepcidin Regulation

In 2012, we observed in vitro that sustained, submicromolar H_2_O_2_ stimulates hepatocellular hepcidin expression through STAT3 and its response element in the hepcidin promoter [45]. This finding was obtained using a glucose oxidase/catalase system (GOX/CAT), which continuously generated low levels of H_2_O_2_ while consuming O_2_ and thereby more closely simulated the continuous production of H_2_O_2_ by cellular oxidases than a single peroxide bolus [47,48]. The results showed that H_2_O_2_ is not merely a product of oxidative damage but can act as a concentration-dependent signaling molecule; excessive peroxide, in contrast, may cause nonspecific oxidative injury and suppress hepcidin [49,50]. Because the GOX/CAT system combines continuous H_2_O_2_ generation with oxygen consumption, it provides a physiologically informed model of endogenous redox signaling, while the resulting hepcidin responses remain experimentally measured cellular responses.

Low oxygen tension further amplifies H_2_O_2_-induced hepcidin expression through STAT3, without requiring direct activation of HIF-1α [46]. Oxygen availability and H_2_O_2_ generation are therefore closely linked within the same redox system. At the cellular level, this oxygen–redox environment may directly influence hepcidin regulation: STAT3 is redox sensitive, and peroxiredoxin-2 can transmit H_2_O_2_-derived oxidative equivalents to STAT3 [51,52]. Erythrocyte-derived heme provides an additional connection, because its degradation by HO-1 releases Fe^2+^, which can interact with H_2_O_2_ through Fenton chemistry. At controlled levels, this interaction may contribute to regulated redox signaling, whereas excessive iron and peroxide promote nonspecific oxidative damage.

**Figure 3:**
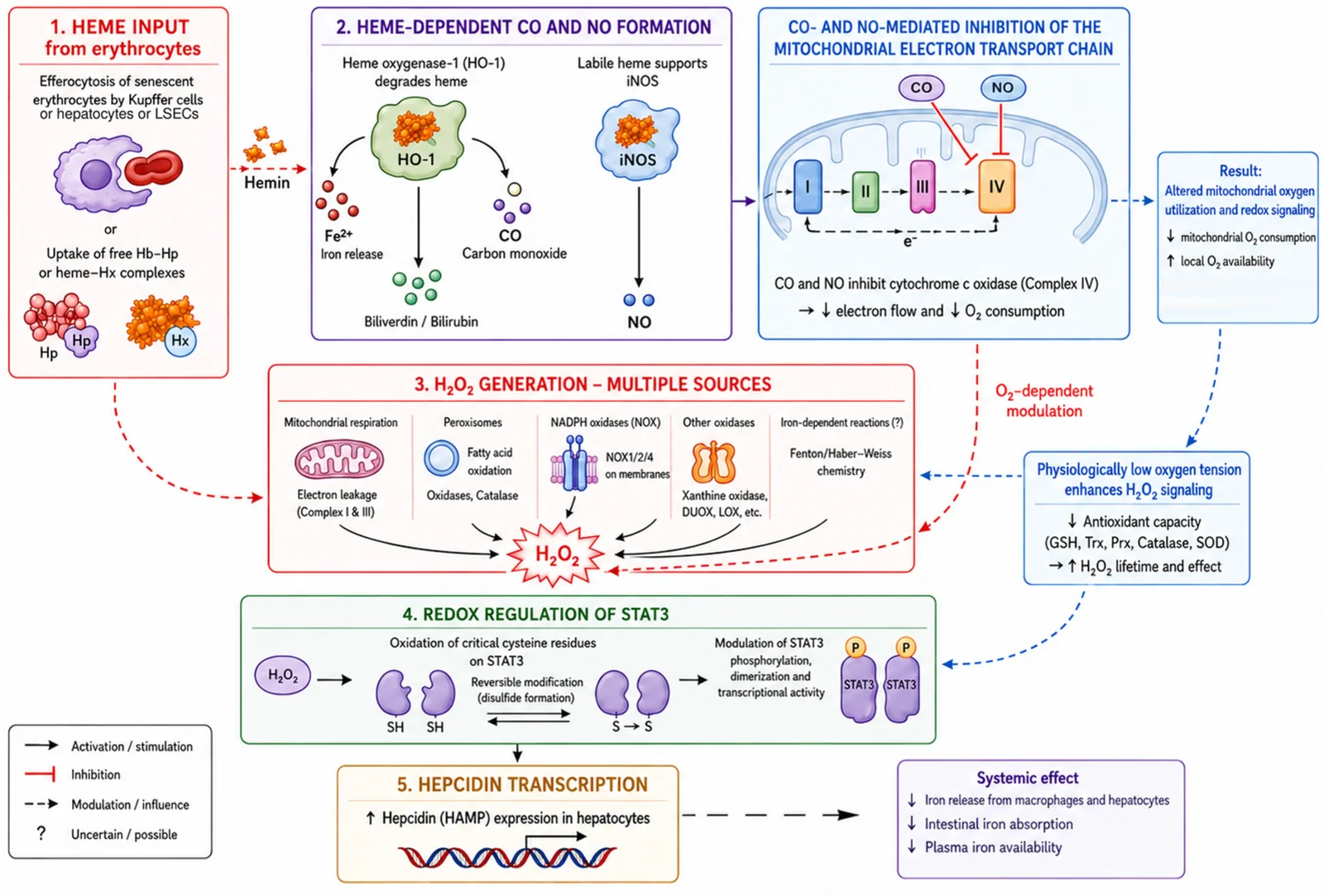
Heme-driven oxygen- and redox-sensitive STAT3 signaling in hepatocellular hepcidin regulation. Erythrocyte turnover links this oxygen–redox system directly to heme metabolism. HO-1-mediated degradation of erythrocyte-derived heme releases Fe^2+^, biliverdin, and carbon monoxide (CO); recycling of approximately 25 mg of erythrocyte iron per day is estimated to generate approximately 10 mL of CO. Heme availability may also influence nitric oxide (NO) formation because nitric oxide synthases require heme, as illustrated by the dependence of macrophage iNOS induction on the labile heme pool [52]. Basal hepatic NO production has been estimated at approximately 5 mL per day [53]. CO and NO can inhibit mitochondrial complex IV, reduce oxygen consumption, increase local oxygen availability, and modify ROS formation. H_2_O_2_ is additionally generated by mitochondria, peroxisomes, NADPH oxidases, and other oxidases, while Fe^2+^ promotes Fenton chemistry. Physiologically low oxygen tension may prolong H_2_O_2_ activity by reducing antioxidant capacity. Together, altered mitochondrial oxygen utilization and sustained H_2_O_2_ signaling may enhance STAT3-dependent hepcidin transcription, thereby restricting cellular iron release, intestinal iron absorption, and plasma iron availability. **Abbreviations:** CO, carbon monoxide; DUOX, dual oxidase; Fe^2+^, ferrous iron; GSH, glutathione; H_2_O_2_, hydrogen peroxide; HAMP, hepcidin antimicrobial peptide; Hb, hemoglobin; HO-1, heme oxygenase-1; Hp, haptoglobin; Hx, hemopexin; iNOS, inducible nitric oxide synthase; LOX, lipoxygenase; NO, nitric oxide; NOX, NADPH oxidase; Prx, peroxiredoxin; SH, reduced cysteine thiol; S–S, oxidized disulfide bond; SOD, superoxide dismutase; STAT3, signal transducer and activator of transcription 3; Trx, thioredoxin.

The scale of this system is considerable. At rest, the liver consumes approximately 60–80 L of O_2_ per day, and historical estimates suggest that 2–3% of this oxygen may give rise to H_2_O_2_ [53]. Although most H_2_O_2_ is rapidly removed by antioxidant systems, its continuous formation establishes a dynamic hepatic redox environment. Thus, hepcidin may be influenced not only by classical systemic signals but also by the basal oxygen–redox conditions prevailing within hepatic cells. This cell-level regulation by oxygen availability and continuous H_2_O_2_ signaling has not yet been fully incorporated into current models of hepcidin regulation. Erythrocyte turnover connects this oxygen–redox environment with hepatic heme metabolism (Figure 3).

Although present in much smaller amounts than O_2_, CO and NO can reversibly inhibit cytochrome c oxidase in a concentration-, redox-, and oxygen-dependent manner, thereby altering mitochondrial respiration, intracellular oxygen distribution, and ROS formation [54,55]. Experimentally, 50 ppm CO reduced complex IV activity in isolated human mitochondria by approximately 20%, whereas NO inhibits cytochrome c oxidase at nanomolar concentrations [56,57]. Direct hepatic evidence comes from cytoglobin-deficient stellate cells, which released NO into neighboring hepatocytes, inhibited cytochrome c oxidase, reduced respiration, and increased ROS formation [58].

Together, these findings support a model in which erythrocyte-derived heme, Fe^2+^, CO, and heme-dependent NO formation interact with mitochondrial respiration, oxygen availability, and H_2_O_2_–STAT3 signaling. Hepcidin may therefore be regulated not only by classical systemic iron and inflammatory signals but also by the local oxygen–redox environment of hepatic cells. The proposed oxygen–heme–gasotransmitter–redox network should thus be viewed as a complementary regulatory layer that may converge on hepcidin regulation, rather than as a fully established linear pathway.

## 7. Unresolved Contradictions in Hepcidin Regulation

The classical model of systemic iron regulation predicts that increasing iron availability induces hepatic hepcidin through the BMP6–SMAD pathway, whereas inflammation activates hepcidin mainly through cytokine-dependent STAT3 signaling [59,60]. Although these pathways are often described separately, efficient STAT3-mediated hepcidin induction during inflammation depends on intact BMP–SMAD signaling [61]. However, several observations do not fit easily into this linear framework. Systemic iron administration in vivo generally increases hepatic hepcidin, whereas direct exposure of isolated hepatocytes to iron or heme often produces different responses. Many in vitro studies use iron concentrations of approximately 50 µM or higher, thereby examining overt intracellular iron stress rather than physiological iron sensing. Under these conditions, hepcidin suppression may reflect excessive cellular iron loading rather than the normal response to increasing iron availability.

A similar bias affects the interpretation of hemolysis. Most studies focus on severe hemolytic diseases or strong experimental hemolysis [62], in which erythropoietic stimulation, erythroferrone release, oxidative injury, liver damage, and high intracellular heme burden favor hepcidin suppression. Mild hemolysis has received less attention, although our preliminary phenylhydrazine-induced hemolysis experiments indicate that it initially induces hepatic hepcidin through an HFE-dependent iron response before stronger or sustained hemolysis shifts the system toward suppression (Mueller, S., manuscript in preparation). In these experiments, STAT3 activation was observed after hemolysis, including in HFE-deficient mice, but this activation was not sufficient to induce the early hepcidin response. These findings suggest that redox-sensitive STAT3 signaling contributes to the hemolysis-associated response but cannot substitute for the HFE-dependent BMP–SMAD framework required for effective hepcidin induction. This distinction may be relevant in alcohol-associated liver disease, in which hemolysis and enhanced erythrocyte turnover have only recently been established [3]. Acute ethanol models demonstrated hepcidin suppression through oxidative stress and impaired C/EBPα-dependent transcription [63], whereas chronically heavy-drinking humans exhibit increased circulating hepcidin [9,24]. In patients with more severe disease, serum hepcidin may decline slightly but remains elevated compared with healthy controls (see also Supplementary Table S1). The focus on acute alcohol exposure and advanced hemolysis may therefore have obscured the biphasic and disease-stage-dependent nature of the response.

Comparable discrepancies concern oxygen and redox signaling. Most hepatocyte experiments are performed at atmospheric oxygen concentrations of 21%, although hepatocytes in vivo are exposed to substantially lower oxygen tensions, generally below approximately 8%, with intracellular oxygen concentrations approaching 2% [48]. Conventional cell culture may therefore alter mitochondrial metabolism, ROS generation, redox buffering, and signal transduction.

HIF-1α is often interpreted as a sensor of an absolute low-oxygen threshold, although experimental evidence indicates that it responds primarily and transiently to decreases in oxygen tension. After adaptation to stable low oxygen, HIF-1α is degraded through the HIF-1α/PHD feedback loop despite continued hypoxia [48]. Thus, the rate and magnitude of oxygen decline may be more relevant than the final oxygen concentration. Systemic hypoxia and local hepatic oxygen limitation should therefore not be considered mechanistically equivalent. Moreover, a functional hypoxia-response element mediating direct HIF-dependent repression of the hepcidin promoter has not been convincingly established [64]. Systemic hypoxia may suppress hepcidin indirectly through EPO-driven erythropoiesis and erythroferrone release, whereas local low oxygen tension in hepatocytes may potentiate redox-sensitive hepcidin signaling. Sustained low-level H_2_O_2_ induces hepcidin through STAT3, and physiologically low oxygen tension enhances this response through, e.g., NOX4-dependent redox signaling independently of direct HIF-1α activation [45,46].

The mode of H_2_O_2_ exposure represents another important methodological issue. Conventional bolus application generates transient and often supraphysiological oxidative stress, whereas H_2_O_2_ is typically generated continuously at very low concentrations over prolonged periods [48,50]. These exposure modes can produce fundamentally different cellular responses. Sustained low-level H_2_O_2_ is therefore more relevant to physiological redox signaling than a single high-dose bolus, particularly when oxygen tension is controlled simultaneously. Earlier studies also demonstrated that catalase activity and H_2_O_2_ removal differ profoundly between intact erythrocytes or hepatocytes and their homogenates, emphasizing the importance of membrane permeability and cellular compartmentalization [65].

Hepcidin regulation is strongly cell-type-dependent. During differentiation of human monocytes into macrophages, macrophage hepcidin increased approximately 60-fold, accompanied by NOX2-dependent H_2_O_2_ formation, reduced ferroportin expression, and increased ferritin expression [66]. Despite this substantial relative induction, basal macrophage hepcidin expression was approximately eightfold lower than hepatocyte hepcidin expression, and macrophage-derived hepcidin accounted for only about 10% of total hepcidin in the macrophage–hepatocyte co-culture model [66]. These findings support a predominantly local or autocrine role of macrophage-derived hepcidin, whereas hepatocyte-derived hepcidin remains the dominant regulator of systemic iron homeostasis. Macrophage hepcidin was inhibited by catalase or NOX blockade and, unlike hepatocyte hepcidin, was not further enhanced by low oxygen tension. Differentiated macrophages nevertheless stimulated hepatocyte hepcidin through IL-1β-dependent activation of STAT3 and CEBPδ, an effect strongly potentiated under physiologically low oxygen conditions.

These apparently conflicting findings therefore arise from both biological complexity and experimental design. The hepcidin response depends on iron concentration and form, exposure duration, oxygen tension and kinetics, redox conditions, hemolysis severity, erythropoietic activity, and the responding cell type. Resolving these variables is essential for integrating systemic iron loading, erythrocyte turnover, redox signaling, and hepatic hepcidin regulation within a unified red blood cell–liver axis.

## 8. The Red Blood Cell–Liver Axis: Erythrocyte Clearance Beyond Macrophages

The classical view of erythrocyte clearance assigns a central role to macrophages, particularly splenic macrophages and hepatic Kupffer cells, as the principal cells responsible for removing senescent or damaged erythrocytes [28]. Recent work has further shown that controlled hemolysis within splenic red-pulp macrophages contributes to physiological erythrocyte turnover [67]. Hemoglobin released during splenic erythrocyte processing may subsequently be cleared by LSECs, supporting a spleen-to-liver pathway for hemoglobin handling [68]. While this paradigm has shaped the field of iron metabolism for decades, experimental evidence now indicates that hepatic erythrocyte processing is considerably more complex and involves multiple liver cell populations.

**Figure 4:**
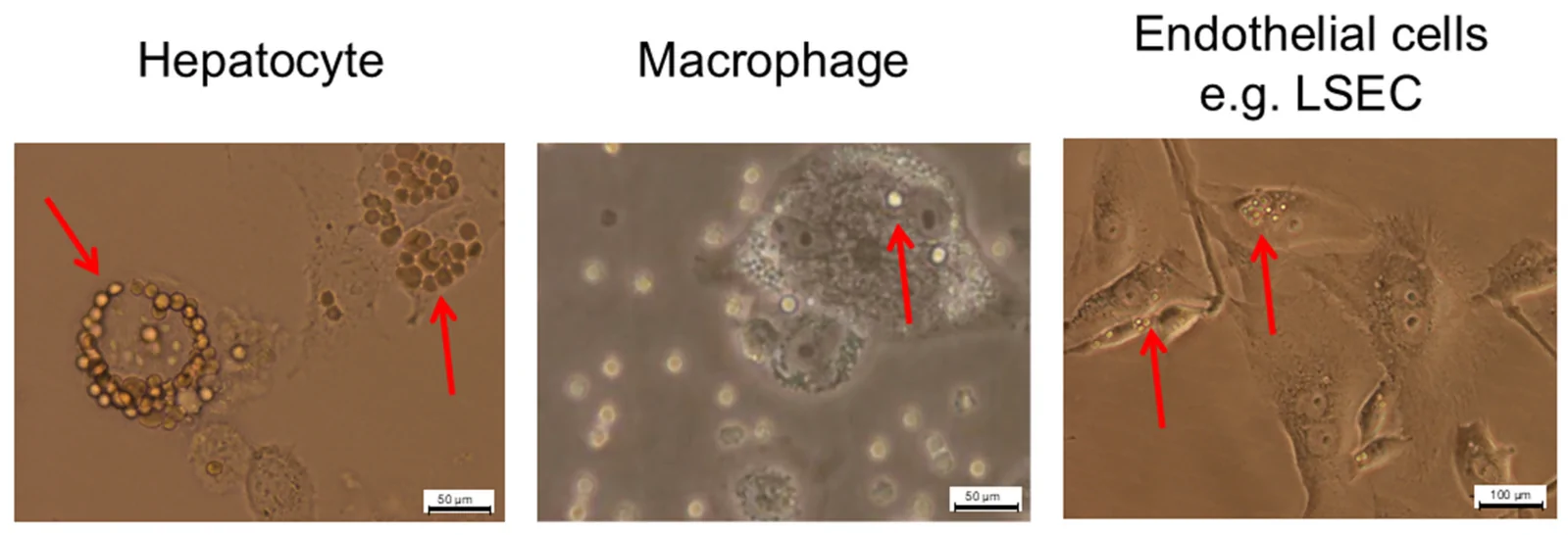
Multiple hepatic cell types participate in erythrocyte clearance following erythrocyte injury. Representative phase-contrast microscopy images showing erythrocyte uptake by hepatocytes, macrophages, and liver sinusoidal endothelial cells (arrows). Oxidative stress, ethanol exposure, and other forms of erythrocyte injury induce eryptosis with phosphatidylserine externalization, generating an “eat-me” signal that promotes erythrophagocytosis and efferocytic uptake. Erythrocyte uptake is observed in all three hepatic cell types [3,4,5]; however, intact internalized RBCs are particularly prominent and persist within hepatocytes. This likely reflects rapid digestion of engulfed RBCs by professional phagocytes, whereas hepatocytes process them less efficiently and, in vitro, lack a fully functional canalicular excretory system. These findings demonstrate that erythrocyte clearance is not restricted to professional phagocytes and support the concept of a multicellular hepatic erythrocyte-processing network linking erythrocyte injury to heme degradation, iron recycling, bilirubin production, and redox signaling. Abbreviations: LSEC, liver sinusoidal endothelial cell.

As illustrated in Figure 4, damaged erythrocytes are processed by a multicellular hepatic network comprising macrophages, liver sinusoidal endothelial cells (LSECs), and hepatocytes. Although the importance of the liver at the organ level was demonstrated as early as the 1940s, the participating hepatic cell types remained incompletely defined. Following acetylphenylhydrazine-induced destruction of radioactively labeled erythrocytes, 46% of the erythrocyte iron disappearing from the circulation accumulated in the liver within six days, compared with only 17% in the spleen [69]. Hepatic radioiron was incorporated into ferritin and subsequently reutilized for hemoglobin synthesis [69,70]. However, these studies did not establish which hepatic cells had taken up the erythrocytes or their iron. Our recent studies provided direct evidence that damaged erythrocytes are internalized not only by macrophages but also by hepatocytes and endothelial-like hepatic cells, thereby establishing the concept of a multicellular hepatic RBC-clearance network [3,4,5]. Asialoglycoprotein receptor 1 contributes to erythrocyte uptake by hepatocytes, while stabilin-dependent recognition represents an important component of the corresponding pathway in LSECs [4,5,71]. More broadly, these findings point to a complex and still incompletely characterized repertoire of hepatic recognition and scavenger receptors that may cooperate in the uptake of damaged erythrocytes and other altered-self material [72].

Macrophages remain an important component of this network. Kupffer cells clear damaged erythrocytes, while acute erythrocyte stress recruits monocyte-derived iron-recycling macrophages through heme-dependent BACH1 degradation and SPI-C induction [73,74,75]. Alcohol-induced erythrocyte damage likewise enhances hepatic erythrophagocytosis [3]. Beyond iron recycling, RBC and hemoglobin uptake can functionally reprogram macrophages through CD163-dependent IL-10 release and NRF2–HO-1-associated responses [76,77]. HO-1-derived CO may contribute to this inflammation-limiting program, generating specialized erythrophagocytes that are not adequately captured by the traditional M1/M2 classification [77,78,79]. However, the long-term viability of splenectomized individuals demonstrates that splenic clearance is not indispensable and can be compensated by other compartments, particularly the liver [80].

**Figure 5:**
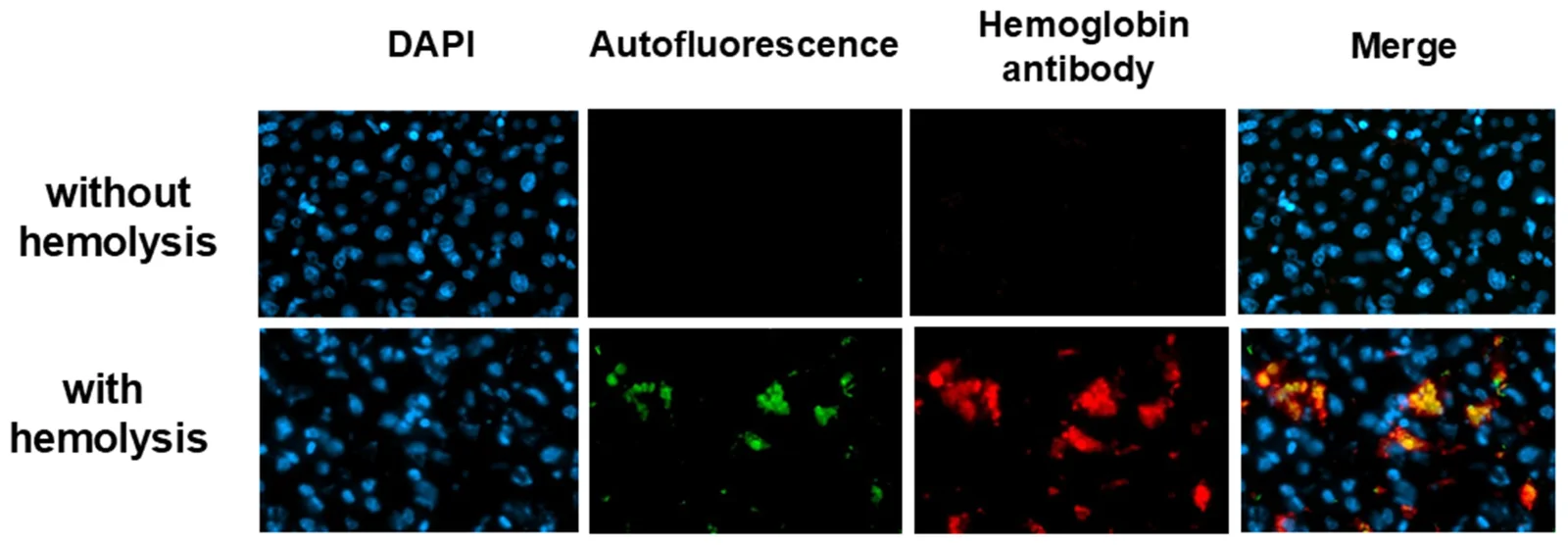
Intrahepatic hemoglobin accumulation in patients with alcohol-associated liver disease with systemic hemolysis. Representative immunofluorescence images of liver biopsies from patients with alcohol-associated liver disease with or without biochemical evidence of hemolysis. Nuclei are shown by DAPI staining, endogenous hemoglobin autofluorescence is shown in green, hemoglobin immunostaining in red, and colocalization in yellow. Patients without hemolysis show minimal hepatic hemoglobin signal, whereas patients with hemolysis show marked intrahepatic hemoglobin accumulation together with elevated ferritin and sCD163 and reduced hemoglobin levels. Of note, hemoglobin was detected in all three hepatic cell populations examined, including LSECs, macrophages, and hepatocytes. These findings provide direct histological evidence that systemic hemolysis increases hepatic exposure to hemoglobin and heme in human ALD. Notably, most of the long-known autofluorescence of liver sections is derived from hemoglobin. Abbreviations: ALD, alcohol-associated liver disease; DAPI, 4′,6-diamidino-2-phenylindole; sCD163, soluble CD163. Reference: [4].

The physiological relevance of these mechanisms is supported by observations in human liver tissue. As shown in Figure 5, patients with alcohol-associated liver disease and biochemical evidence of enhanced hemolysis exhibit marked intrahepatic hemoglobin accumulation, demonstrated by both hemoglobin autofluorescence and immunofluorescence staining [3,4]. These findings indicate that erythrocyte-derived material reaches the liver in vivo and accumulates within hepatic tissue under pathological conditions, providing direct histological evidence for an active red blood cell–liver axis. Notably, Figure 5 also demonstrates that most of the long-known autofluorescence of liver sections is derived from hemoglobin.

Once internalized, erythrocytes enter a common hepatic processing pathway centered on heme degradation by heme oxygenase-1 (HO-1) [78,79]. Intravascular hemolysis provides an alternative route by releasing hemoglobin, heme, and iron into the circulation. Early studies demonstrated that circulating hemoglobin is taken up and degraded within hepatocellular lysosomes and that hemoglobin-derived iron accumulates in hepatic parenchymal cells [81,82]. These soluble products are delivered through distinct carrier and receptor systems: hemoglobin binds to haptoglobin and is internalized through CD163, heme binds to hemopexin and is taken up through CD91/LRP1, and transferrin-bound iron is internalized through transferrin receptor-1 [83,84]. Under conditions of iron overload, when transferrin-binding capacity is exceeded, hepatocytes can also take up non-transferrin-bound iron (NTBI), in part through ZIP14 (SLC39A14) [85]. In addition to intact erythrocytes [5] and soluble hemolysis-derived molecules [10], RBC-derived extracellular vesicles represent another potentially important route by which erythrocyte material reaches the liver. During their lifespan, erythrocytes shed hemoglobin-containing, phosphatidylserine-exposing vesicles that are rapidly cleared from the circulation, predominantly by hepatic Kupffer cells through scavenger receptor-dependent mechanisms [27]. This vesicular pathway may continuously deliver hemoglobin, heme, iron, membrane lipids, and redox-active cargo to the liver and may become amplified during oxidative stress, eryptosis, and liver disease. Thus, erythrophagocytosis, efferocytic uptake, hemolysis, and extracellular vesicle release constitute complementary routes by which erythrocyte-derived material is delivered to and processed by the liver. Electron microscopy further suggests close structural interactions between erythrocyte-containing intracellular compartments and mitochondria [7] (see electron micrographs in the Appendix, pp. 1461–1462). Although direct membrane fusion remains unproven, such contact sites may facilitate the transfer of erythrocyte-derived heme, iron, membrane lipids, and redox signals to mitochondria, thereby potentially linking hepatic erythrocyte uptake to mitochondrial dysfunction, lipid peroxidation, and ferroptotic injury.

**Figure 6:**
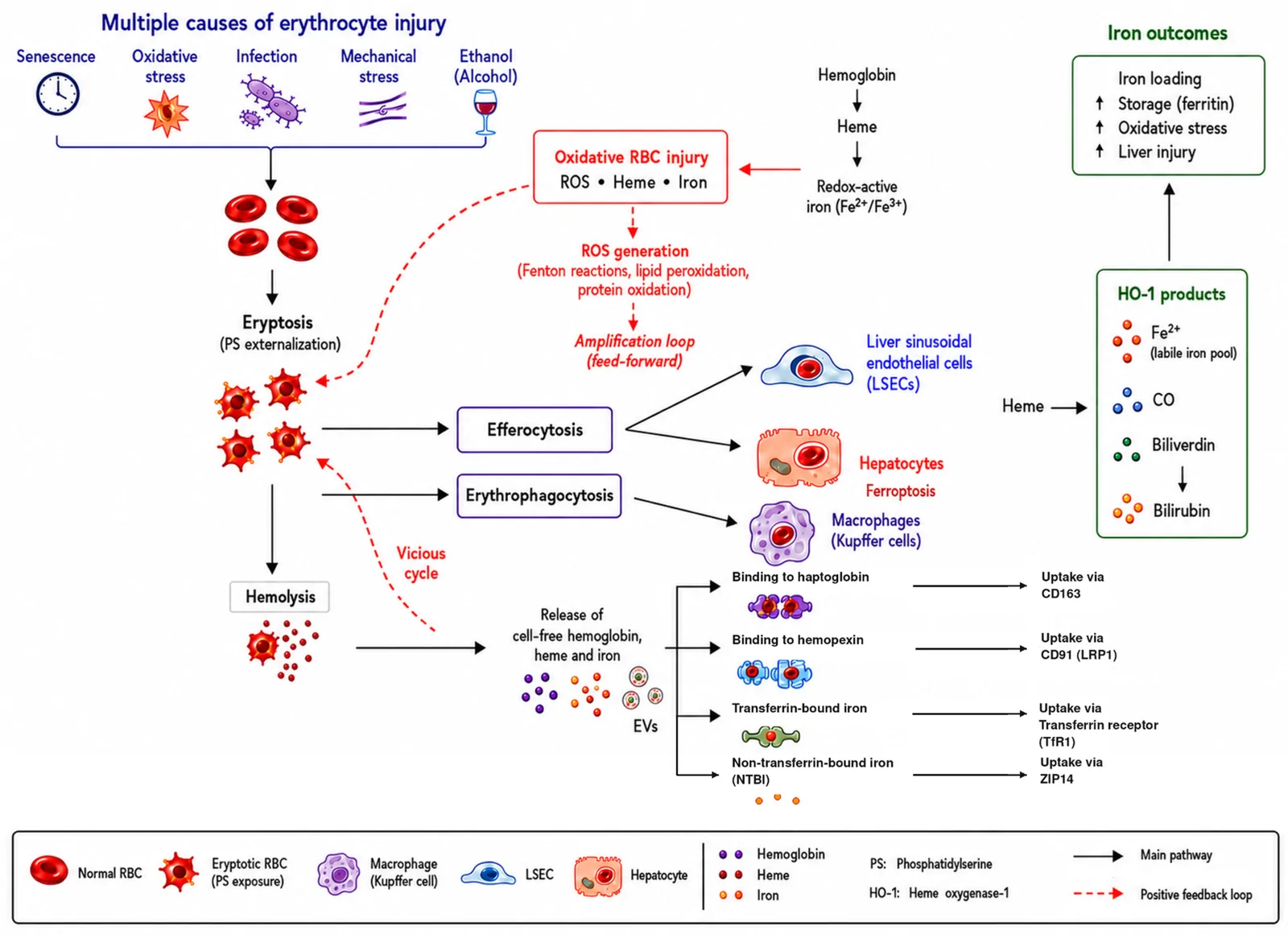
Diverse causes of erythrocyte injury converge on hepatic heme processing and iron signaling. Physiological erythrocyte senescence and pathological stimuli, including oxidative stress, infection, mechanical stress, ethanol exposure, and hemolytic disorders, generate a continuum from eryptosis to intravascular hemolysis. During eryptosis, phosphatidylserine externalization promotes efferocytosis and erythrophagocytosis by macrophages, hepatocytes, and LSECs. RBC-derived extracellular vesicles provide an additional route for delivering hemoglobin, heme, iron, and membrane lipids to the liver. Internalized erythrocytes undergo heme degradation by HO-1, generating ferrous iron, carbon monoxide, and biliverdin. In parallel, intravascular hemolysis releases hemoglobin, heme, and iron, which are scavenged by haptoglobin, hemopexin, and transferrin. Under conditions of iron overload, non-transferrin-bound iron (NTBI) may also arise and be taken up by hepatocytes, in part via ZIP14 (SLC39A14). These complementary pathways link erythrocyte injury to hepatic iron homeostasis, redox signaling, bilirubin production, lipid peroxidation, ferroptotic injury, and liver disease. As highlighted by the red dashed arrows, hemoglobin-derived heme, iron, and bilirubin can themselves promote eryptosis, potentially contributing to a self-amplifying cycle of erythrocyte injury and hepatic heme burden [3]. Abbreviations: PS, phosphatidylserine; ROS, reactive oxygen species; HO-1, heme oxygenase-1; Fe^2+^, ferrous iron; CO, carbon monoxide; CD163, hemoglobin–haptoglobin scavenger receptor; CD91 (LRP1), low-density lipoprotein receptor-related protein 1; TfR1, transferrin receptor-1; LSEC, liver sinusoidal endothelial cell; EVs, extracellular vesicles; NTBI, non-transferrin-bound iron; ZIP14, Zrt- and Irt-like protein 14.

The integrative model proposed in Figure 6 summarizes these pathways. Multiple physiological and pathological stimuli, including senescence, oxidative stress, infection, mechanical stress, and ethanol exposure, converge on common mechanisms of erythrocyte injury. Eryptosis, erythrophagocytosis, efferocytic uptake, and hemolysis are therefore best viewed as interconnected components of a unified biological program rather than isolated processes. Importantly, products generated during erythrocyte breakdown, including free heme, iron, and bilirubin [3], as well as accumulating bile acids [86], can themselves promote further erythrocyte injury and eryptosis, thereby potentially establishing a self-amplifying vicious cycle [3,9]. In advanced liver disease, the enhanced hemolysis observed in patients with cirrhosis may therefore result not only from mechanical shear stress and altered erythrocyte membrane properties but also from the accumulation of bilirubin, bile acids, heme, iron, and other redox-active metabolites [3,9].

Through this reciprocal interaction, hepatic processing of erythrocyte-derived material provides a novel mechanistic framework linking erythrocyte turnover to iron homeostasis, oxidative signaling, inflammation, fibrosis, and ultimately clinical outcomes.

## 9. A Biphasic Hepcidin Response to Erythrocyte-Derived Heme and Iron

The apparently conflicting effects of iron, heme, hemolysis, hypoxia, and inflammation on hepcidin suggest that hepatic iron sensing is strongly context-dependent. Hepcidin regulation is determined not by iron availability alone, but by the molecular form and magnitude of iron delivery, the route by which erythrocyte-derived material reaches the cell, the responding cell type, and the prevailing redox, oxygen, inflammatory, and erythropoietic environment. Although uptake of intact erythrocytes, hemoglobin–haptoglobin complexes, heme–hemopexin complexes, and non-heme iron represents distinct pathways, these routes ultimately converge on intracellular heme and iron processing. They are nevertheless not mechanistically equivalent, because intracellular heme must first be degraded by HO-1, generating ferrous iron, carbon monoxide, and biliverdin, whereas non-heme iron enters the intracellular iron pool without this preceding reaction.

**Figure 7:**
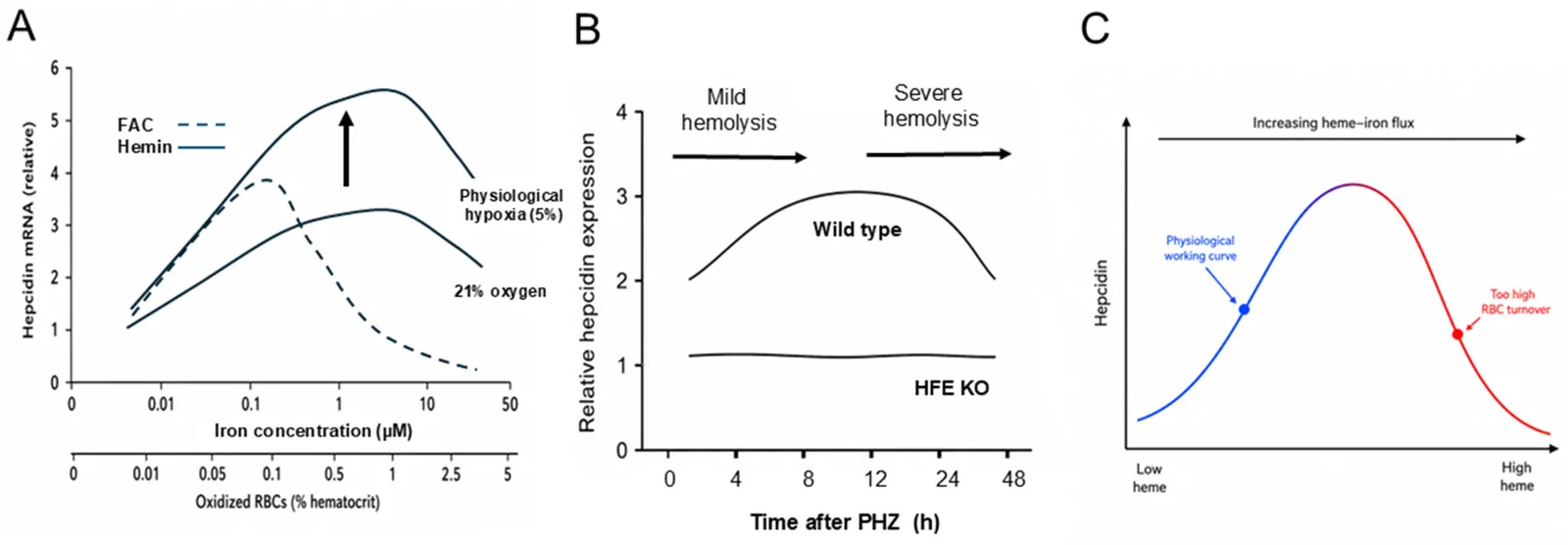
Biphasic hepatic hepcidin regulation by erythrocyte-derived heme and iron: in vitro, in vivo, and proposed mechanism. (**A**). Biphasic regulation of hepatocellular hepcidin by iron, heme, and physiological hypoxia. Schematic summary of biphasic hepcidin regulation in hepatocytes. Low-to-moderate concentrations of iron or heme stimulate hepcidin expression, whereas excessive exposure attenuates or suppresses the response. Ferric ammonium citrate induces a transient response with an early peak and rapid decline, whereas hemin produces a stronger and more sustained induction. Physiological hypoxia further potentiates hemin-induced hepcidin expression while preserving the biphasic dose–response pattern. A quantitative comparison suggests that soluble hemin and oxidized erythrocyte uptake may generate a similar effective intracellular heme burden despite different experimental designs. Distinct heme-responsive genes, including NRF2, ferritin, hepcidin, and HO-1, display different activation thresholds, indicating that hepatocytes integrate erythrocyte-derived heme signals over a broad dynamic range. (**B**). HFE-dependent hepatic hepcidin response during phenylhydrazine-induced hemolysis in mice. Schematic representation of hepatic hepcidin expression after phenylhydrazine-induced hemolysis in wild-type and Hfe-knockout mice. In wild-type mice, mild hemolysis induces a transient increase in hepatic hepcidin expression, peaking between 8 and 24 h and declining thereafter as hemolysis becomes more severe or sustained. In contrast, Hfe-knockout mice fail to mount a comparable early hepcidin response, indicating that HFE is required for the hepatic sensing or translation of hemolysis-derived iron signals into hepcidin induction. Although STAT3 activation is retained after hemolysis, it is insufficient to compensate for the loss of HFE-dependent sensing and the associated BMP–SMAD-dependent hepcidin-regulatory framework. The scheme illustrates the biphasic concept in vivo: mild hemolysis stimulates hepcidin, whereas stronger hemolytic stress shifts the system toward attenuation or suppression. Data are presented schematically; manuscript in preparation. (**C**). Proposed mechanism of the biphasic hepatocellular regulation of hepcidin. The schematic illustrates that increasing iron and heme concentrations initially induce hepcidin expression in hepatocytes in vitro. A comparable stimulatory effect has been observed in hepatocyte–endothelial cell co-culture systems and was associated with additional endothelial BMP6 secretion [9,87]. This hepcidin induction is enhanced by H_2_O_2_ and hypoxia and can be partially inhibited by N-acetylcysteine [45,46]. Similar H_2_O_2_-dependent effects have also been described in macrophages, although without an apparent dependence on hypoxia [66]. Only at high iron and heme concentrations does hepcidin expression become suppressed. Mechanistically, this may involve alterations in cellular redox buffering through modulation of the STAT3 pathway; further molecular studies are required. At very high concentrations, cytotoxicity, including apoptotic cell injury, may additionally contribute. This mechanism may complement ERFE-mediated hepcidin suppression, as the original description of ERFE showed that its deletion markedly attenuated, but did not completely abolish, erythropoiesis-induced hepcidin suppression [88].

As illustrated in Figure 7A, preliminary experimental data from our laboratory, previously presented in a concept review, indicate that hepatocytes display a biphasic response to increasing concentrations of iron and heme [9]. Low physiological concentrations stimulate hepcidin expression, whereas higher concentrations progressively attenuate or suppress this response. This regulation depends on the biochemical form of iron: ferric ammonium citrate (FAC) induces a transient response with an early peak followed by rapid decline, whereas hemin produces a stronger and more sustained induction. Physiological hypoxia (5% O_2_) further potentiates the hemin response while preserving the biphasic dose–response pattern [46,87], suggesting that hepatocytes sense not only iron abundance but also its molecular form and the surrounding oxygen tension.

Although exposure to soluble hemin and uptake of intact erythrocytes represent fundamentally different experimental systems, both ultimately deliver intracellular heme to hepatocytes. Following cellular uptake, heme is degraded by HO-1, releasing iron that enters the intracellular iron pool. The relevant common denominator is therefore the intracellular delivery and processing of erythrocyte-derived heme rather than the extracellular route by which it reaches the hepatocyte. This provides a direct mechanistic link between erythrocyte recycling and hepatocellular iron sensing.

Different heme-responsive genes appear to exhibit distinct activation thresholds. NRF2 responds at very low erythrocyte-derived heme concentrations, consistent with its early activation during oxidative stress, while ferritin is induced similarly early, reflecting rapid intracellular iron sequestration through the IRE/IRP network [5]. Hepcidin requires a greater heme flux for maximal induction, whereas HO-1 shows a dose-dependent response that declines again at high erythrocyte or heme burden. These findings indicate that hepatocytes decode erythrocyte-derived signals across a broad dynamic range rather than through a simple linear sensing mechanism. Experimental and clinical evidence further supports that increasing heme, iron, and oxidative toxicity actively contributes to hepcidin attenuation and suppression at high exposure levels [9,45]. Of note and as shown in Figure 7A, hypoxia shifts the hepcidin-biphasic response to iron/heme levels to higher levels [46]. The biphasic concept is further supported by preliminary in vivo observations in a phenylhydrazine (PHZ)-induced hemolysis model (Figure 7B; manuscript in preparation).

The endothelial BMP6 signal is proposed to act through hepatocellular BMP receptor–SMAD signaling and to contribute primarily to the ascending stimulatory limb of the biphasic response, whereas HFE is considered an essential component of the upstream hepatic iron-sensing mechanism. Accordingly, I do not propose a distinct HFE-independent mechanism; rather, HFE-dependent sensing is considered to converge on, or to be required for, an effective BMP6–SMAD-mediated transcriptional response. The proposed mechanism is further illustrated by the phenylhydrazine-induced hemolysis model (Figure 7B; manuscript in preparation). The accompanying activation of STAT3 after phenylhydrazine-induced hemolysis suggests that redox-sensitive STAT3 signaling also contributes to the hemolysis-associated response; however, STAT3 activation alone was insufficient to produce the early hepcidin increase in HFE-deficient mice.

Following mild hemolysis, wild-type mice exhibited rapid transient induction of hepatic hepcidin, whereas HFE-deficient mice failed to mount this early response despite exposure to the same hemolytic stimulus. This suggests that the initial physiological hepcidin response requires intact HFE-dependent iron sensing. Recent work showing that HFE requires TFR2 for early hepcidin induction after an acute dietary iron challenge is consistent with this HFE dependence, although the role of TFR2 during hemolysis remains unknown [89]. More severe or sustained hemolysis was subsequently associated with hepcidin suppression, consistent with the descending arm of the proposed biphasic response.

Importantly, the biphasic response is already evident at the level of the hepatocyte and therefore does not necessarily require an additional erythroid suppressor such as erythroferrone (ERFE). Low intracellular heme and iron burdens stimulate hepcidin, whereas excessive exposure directly attenuates or suppresses this response. In vivo, however, stronger or sustained hemolysis also stimulates erythropoiesis and activates the EPO–ERFE axis, which further suppresses hepcidin through inhibition of hepatic BMP–SMAD signaling [88]. Consistent with this mechanism, PHZ-induced hemolytic anemia caused a rapid increase in circulating EPO and erythroid ERFE expression, followed by progressive hepatic hepcidin suppression, which was significantly attenuated by ERFE knockdown [62]. ERFE should therefore be regarded as an important systemic amplifier of hepcidin suppression rather than as the sole explanation for the descending arm of the biphasic response. Indeed, ERFE deficiency markedly attenuates but does not completely abolish hepcidin suppression following erythropoietic stimulation, supporting the existence of additional ERFE-independent mechanisms as reported earlier [90].

These observations also provide an attractive explanation for basal hepcidin expression. Continuous physiological erythrocyte turnover delivers heme and iron to the liver every day and may therefore generate a persistent HFE-dependent stimulatory signal that contributes to the basal hepcidin set point. This model predicts that loss of this component in HFE deficiency would lower basal hepcidin without necessarily abolishing all remaining regulatory inputs. This may help explain why HFE-associated hemochromatosis shows variable and often limited clinical penetrance as low as 20% [91]: the phenotypic consequences of impaired HFE-dependent sensing may depend on the magnitude of erythrocyte turnover, compensatory BMP6–SMAD signaling, inflammatory tone, erythropoietic demand, and other modifiers of the basal hepcidin response. It may also explain why alcohol consumption and the associated increase in heme turnover can markedly modify hepcidin regulation. Alcohol-induced hemolysis and enhanced erythrocyte turnover have only recently been recognized and have therefore not been systematically incorporated into clinical studies of hepcidin regulation [3,24] (see also Table 1).

Together, the cellular dose–response data and the in vivo hemolysis model support a two-level concept: a primary biphasic hepatocellular response to erythrocyte-derived heme and iron, superimposed by systemic erythropoietic suppression through EPO, ERFE, and related pathways during more severe or sustained hemolysis. Physiological erythrocyte-derived heme and iron initially stimulate hepcidin, whereas excessive erythrocyte turnover progressively shifts the system toward suppression.

## 10. The Central Role of Erythrocyte Turnover in Systemic Iron Homeostasis

**Figure 8:**
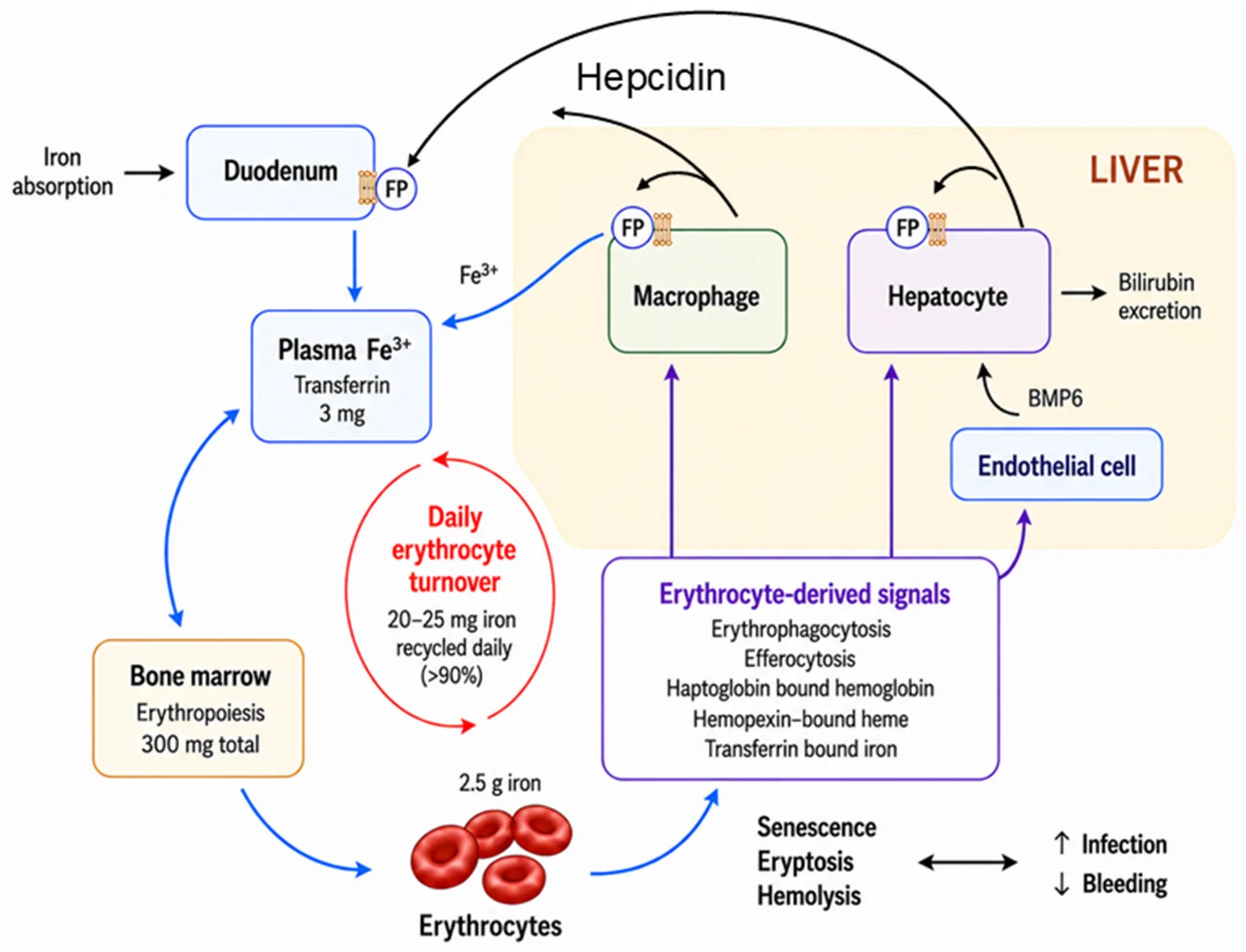
The red blood cell–liver axis as the central regulator of systemic iron homeostasis. Schematic illustration of the concept that continuous erythrocyte turnover is the major physiological source of iron- and heme-derived signals regulating hepatic hepcidin expression. Approximately 20–25 mg of iron is recycled daily through erythrocyte senescence, eryptosis, and physiological hemolysis, whereas only 1–2 mg is absorbed from the intestine. Erythrocyte-derived products reach the liver through erythrophagocytosis by Kupffer cells, efferocytosis by hepatocytes, and uptake of haptoglobin-bound hemoglobin, hemopexin-bound heme, and transferrin-bound iron. Hepatocytes integrate these signals with BMP/SMAD, inflammatory, redox, and erythropoietic pathways to regulate hepcidin and coordinate systemic iron availability.

The central concept of this article is not that erythrocyte iron recycling is quantitatively important, which is well established, but that the continuous flux of erythrocyte-derived heme and iron may be processed by a broader hepatic multicellular network and integrated with pathways regulating systemic iron homeostasis. This network includes macrophages, liver sinusoidal endothelial cells (LSECs), and hepatocytes. Erythrocyte turnover is the dominant source of iron reaching the liver (Figure 8). Although historical studies established hepatic iron recycling alongside splenic recycling, they did not determine whether intact erythrocytes were cleared directly by hepatocytes or whether erythrocyte-derived iron was released elsewhere before hepatic storage [69,70].

The physiological relevance of the biphasic hepcidin response described above (see also Figure 7) is supported by complementary in vivo studies using phenylhydrazine-induced hemolysis (manuscript in preparation). Mild hemolysis produced a marked increase in hepatic hepcidin expression, whereas more severe hemolysis induced an initial hepcidin response followed by progressive suppression as anemia and erythropoietic activity developed. Strikingly, the early hepcidin response was completely absent in HFE-deficient mice despite preserved STAT3 activation and continued hepatic iron accumulation. These findings indicate that the early response to erythrocyte-derived iron requires intact HFE-dependent iron sensing, whereas the later suppressive phase reflects the increasing dominance of systemic erythropoietic demand [88].

Rather than representing independent biological processes, erythrocyte senescence, eryptosis, erythrophagocytosis, efferocytosis, and intravascular hemolysis should be viewed as interconnected routes within a continuous erythrocyte turnover process [26,86]. Under physiological conditions, controlled hepatic clearance contributes to the recycling of erythrocytes and their components while limiting extracellular exposure to hemoglobin, heme, and iron. With increasing erythrocyte injury, clearance and scavenging capacity may be exceeded, while hemolysis increases the extracellular release of these products. Diverse disturbances, including bleeding, inflammation, infection, oxidative stress, alcohol exposure, mechanical injury, hemolytic disorders, and ineffective erythropoiesis, can alter erythrocyte turnover, systemic erythropoietic demand, or the amount and nature of erythrocyte-derived material delivered to hepatic cells.

In conclusion, the liver integrates erythrocyte-derived heme and iron with BMP-SMAD signaling, inflammatory cytokines, ERFE-mediated erythropoietic feedback, oxygen tension, and redox pathways to regulate hepcidin. In this dynamic RBC–liver network (Supplementary Figure S3), erythrocyte turnover provides the dominant physiological input, while hemolysis, bleeding, and ineffective erythropoiesis are major modulators.

## 11. The RBC–Liver Axis in Basal Hepcidin Regulation, Hemochromatosis, Infection, Blood Loss, and Hemolytic Anemia

The biphasic model illustrated in Figure 7 provides a common framework for several classical disorders of iron homeostasis. Hepcidin regulation depends not only on iron stores and erythropoietic demand but also on erythrocyte mass and the quantity and biological form of erythrocyte-derived material reaching the liver. Normal erythrocyte turnover generates a continuous basal heme–iron flux that may help establish basal hepcidin expression, positioning the physiological steady state on the lower ascending limb. Moderate increases in erythrocyte turnover and hepatic heme–iron flux may further stimulate hepcidin, whereas very high erythrocyte flux, redox-dependent mechanisms, and increasing erythropoietic demand shift the system toward hepcidin suppression [26,86,88,92].

HFE-associated hemochromatosis provides indirect support for this concept by illustrating defective hepatic sensing of physiological erythrocyte flux. Although normal erythrocyte turnover and basal heme–iron delivery persist, their conversion into an adequate hepcidin signal appears impaired. Hepatocyte-specific Hfe deletion reproduces the systemic iron overload and hepatic hepcidin deficiency of complete Hfe loss, whereas deletion in enterocytes or macrophages does not disturb systemic iron homeostasis [93]. In HFE-deficient mice, the hepcidin response to mild and severe phenylhydrazine-induced hemolysis was absent despite continued hepatic iron accumulation (manuscript in preparation). Patients with HFE-associated hemochromatosis likewise show inappropriately low hepcidin relative to their iron burden and a blunted response to oral iron [94,95]. HFE deficiency may therefore disconnect physiological erythrocyte-derived iron flux from basal and inducible hepcidin expression. Differences in erythrocyte turnover, blood loss, erythropoietic demand, inflammation, and redox signaling may contribute to the variable clinical penetrance of homozygous HFE C282Y [91].

Infection and inflammation predominantly move the system upward along the ascending limb shown in Figure 7. IL-6–JAK–STAT3 signaling directly stimulates the hepcidin promoter, while infection also alters tissue oxygen availability, increases H_2_O_2_ and other redox signals, and promotes erythrocyte membrane damage, eryptosis, and hemolysis [43,49,86]. This increases the delivery of damaged erythrocytes, hemoglobin, heme, and iron to macrophages, LSECs, and hepatocytes [2,3,4,5]. The resulting heme–iron flux and sustained redox signaling may reinforce endothelial BMP6–SMAD signaling and hepatocellular STAT3 activation [45,51,87]. Infection-associated hypoferremia may therefore reflect the combined effects of inflammatory cytokines, altered oxygen and H_2_O_2_ signaling, and increased hepatic exposure to erythrocyte-derived material.

Blood loss moves the system leftward and downward along the ascending limb (see also Figure 7C). As erythrocyte mass and hemoglobin decline, less erythrocyte-derived heme and iron reaches the liver, weakening the physiological signal that supports basal hepcidin expression. At the same time, anemia and tissue hypoxia stimulate erythropoietin production, erythropoiesis, and ERFE-dependent hepcidin suppression [88]. The fall in hepcidin after bleeding can therefore be explained by reduced hepatic stimulation together with increased inhibitory signaling from the erythroid compartment. The graphical representation in Figure S3 is intended as a conceptual description of the combined effects of reduced erythrocyte-derived hepatic stimulation and increased erythroid suppression; the relative contributions of these mechanisms remain experimentally testable.

Severe hemolytic anemias represent the descending limb of the biphasic response shown in Figure 7. In sickle cell disease, hereditary spherocytosis, glucose-6-phosphate dehydrogenase deficiency, autoimmune hemolytic anemia, and thalassemia, accelerated erythrocyte destruction increases the delivery of erythrocytes, hemoglobin, heme, and iron to the liver. At moderate levels, this increased flux may stimulate hepcidin; with increasing severity, however, anemia, hypoxia, oxidative stress, and compensatory or ineffective erythropoiesis increasingly suppress it. Hepcidin therefore falls despite continued or increasing hepatic heme–iron flux [26,86,92].

Thalassemia is a particularly clear example of this descending limb. Ineffective erythropoiesis causes marked expansion of erythroid precursors and strong ERFE-dependent hepcidin suppression, while erythrocyte destruction and delivery of erythrocyte-derived heme and iron to the liver remain increased [88]. As the disease progresses, anemia, hypoxia, and ineffective erythropoiesis outweigh the hepcidin-inductive effects present at lower erythrocyte flux. Hepcidin consequently falls despite increasing intestinal iron absorption and hepatic iron loading.

When hemolysis exceeds hepatic clearance and processing capacity, extracellular hemoglobin and heme additionally promote oxidative injury, inflammation, disturbed iron distribution, and pathological iron accumulation [2,26]. The position of an individual disorder on the biphasic curve therefore depends on the balance between erythrocyte destruction, hepatic clearance, intracellular heme–iron processing, anemia, hypoxia, erythropoietic activity, and transfusional iron loading.

Figure 7 thus places several apparently opposing clinical conditions within one biphasic RBC–liver model. HFE-associated hemochromatosis reflects defective sensing of physiological erythrocyte flux. Infection increases hepcidin through cytokine-, oxygen-, H_2_O_2_-, redox-, and hemolysis-dependent signals on the ascending limb. Blood loss lowers hepcidin by reducing physiological erythrocyte turnover and by increasing ERFE-mediated suppression. Severe hemolytic anemias, particularly thalassemia, move the system beyond the peak onto the descending limb, where hepcidin falls despite continued or increasing hepatic heme–iron flux.

## 12. Hemolysis Beyond Alcohol: A Common Feature in Diverse and Advanced Liver Diseases

While alcohol-associated liver disease provides a particularly informative model of accelerated erythrocyte turnover, disturbed erythrocyte homeostasis may also occur in other chronic liver diseases. Oxidative stress, inflammation, altered lipid metabolism, iron dysregulation, and impaired antioxidant defenses can promote eryptosis, erythrophagocytosis, and hemolysis [3,86]. Increased erythrocyte turnover may therefore represent a common downstream consequence of several apparently unrelated liver disorders rather than a phenomenon specific to alcohol exposure.

MASLD provides an important parallel model. Metabolic and lipid abnormalities may destabilize erythrocyte membranes, promote oxidative injury and eryptosis, and accelerate erythrocyte clearance. Erythrocyte accumulation and erythrophagocytosis by Kupffer cells have been demonstrated in experimental steatohepatitis and human MASLD/NASH and linked to hepatic oxidative stress, inflammation, and fibrosis [96]. Because hepatocytes and LSECs can also internalize erythrocytes, enhanced hepatic processing may contribute to the elevated ferritin levels and hepatic iron accumulation observed in MASLD [97]. Ferritin levels, however, remain higher in alcohol-associated liver disease than in MASLD matched for sex, age, and fibrosis stage [98]. These findings support a potential link between lipid-associated erythrocyte injury, increased RBC turnover, intrahepatic hemoglobin and iron exposure, and progressive liver damage.

Wilson disease illustrates another mechanism. Copper accumulation can promote erythrocyte oxidative injury, impaired antioxidant defense, and membrane damage, and hemolytic episodes may occur particularly during acute or severe disease [99,100]. Anemia and liver injury in Wilson disease may additionally involve erythrocyte damage pathways linked to acid sphingomyelinase and ceramide signaling [101]. More generally, ethanol, copper, reactive oxygen species, inflammatory mediators, and lipid peroxidation products can promote phosphatidylserine exposure, erythrocyte fragility, and enhanced clearance. Impaired antioxidant protection may further increase erythrocyte susceptibility to oxidative injury [102].

Particularly strong support for this concept comes from advanced cirrhosis. In our studies, markers of hemolysis increased with disease severity across different etiologies of chronic liver disease, suggesting that progressive cirrhosis is associated with accelerated erythrocyte destruction irrespective of the initiating cause [3,24]. In patients with alcohol-associated liver disease, serum sCD163, total bilirubin, and indirect bilirubin increased across fibrosis stages, whereas hemoglobin declined (Supplementary Figure S4) [3]. Increased erythrocyte destruction thereby delivers additional hemoglobin, heme, iron, membrane lipids, and bilirubin precursors to the liver. This exposure may influence hepcidin regulation, iron accumulation, oxidative stress, macrophage activation, endothelial dysfunction, fibrosis, and potentially ferroptotic injury. Preliminary data in heavy drinkers further suggest an association between markers of RBC turnover and ferroptosis [103].

This interaction may become self-amplifying. As hepatic dysfunction advances, bilirubin, bile acids, heme, iron, and other redox-active metabolites may further destabilize erythrocytes and promote eryptosis and hemolysis [3,9,86]. Enhanced erythrocyte turnover then increases the hepatic burden of erythrocyte-derived material, potentially aggravating oxidative and inflammatory injury. A similar mechanism may contribute to rapidly progressive syndromes such as severe alcohol-associated hepatitis, fulminant Wilson disease, and acute-on-chronic liver failure, in which profound hepatic dysfunction and accumulation of circulating toxic metabolites may amplify erythrocyte injury [104,105,106,107,108]. Although this concept requires systematic testing, accelerated erythrocyte turnover may be more than a consequence of advanced liver disease and could contribute to its progression.

Taken together, alcohol exposure, copper toxicity, oxidative stress, and cirrhosis converge on shared pathways involving eryptosis, erythrophagocytosis, hemolysis, and hepatic processing of erythrocyte-derived material. This convergence and the potential reciprocal amplification between erythrocyte injury and hepatic dysfunction support the concept of a unified red blood cell–liver axis extending beyond alcohol-associated liver disease.

## 13. Multicellular Heme–Iron–Redox Integration and Gasotransmitters in Hepcidin Regulation

Despite major advances in hepcidin research, a fundamental question remains unresolved: how are iron and heme actually sensed within the liver? The canonical downstream pathway is well established. Endothelial cells, particularly liver sinusoidal endothelial cells (LSECs), produce BMP6, which activates BMP receptors and SMAD signaling in hepatocytes to induce hepcidin [42]. However, neither BMP6 nor its receptors explain how endothelial cells initially detect iron or heme. A similar gap exists in hepatocytes, which respond directly to intracellular heme and iron while integrating endothelial BMP6 input. We propose that redox-sensitive signaling upstream of BMP6 and at the hepcidin promoter may form an important part of this still incompletely defined sensing mechanism.

**Figure 9:**
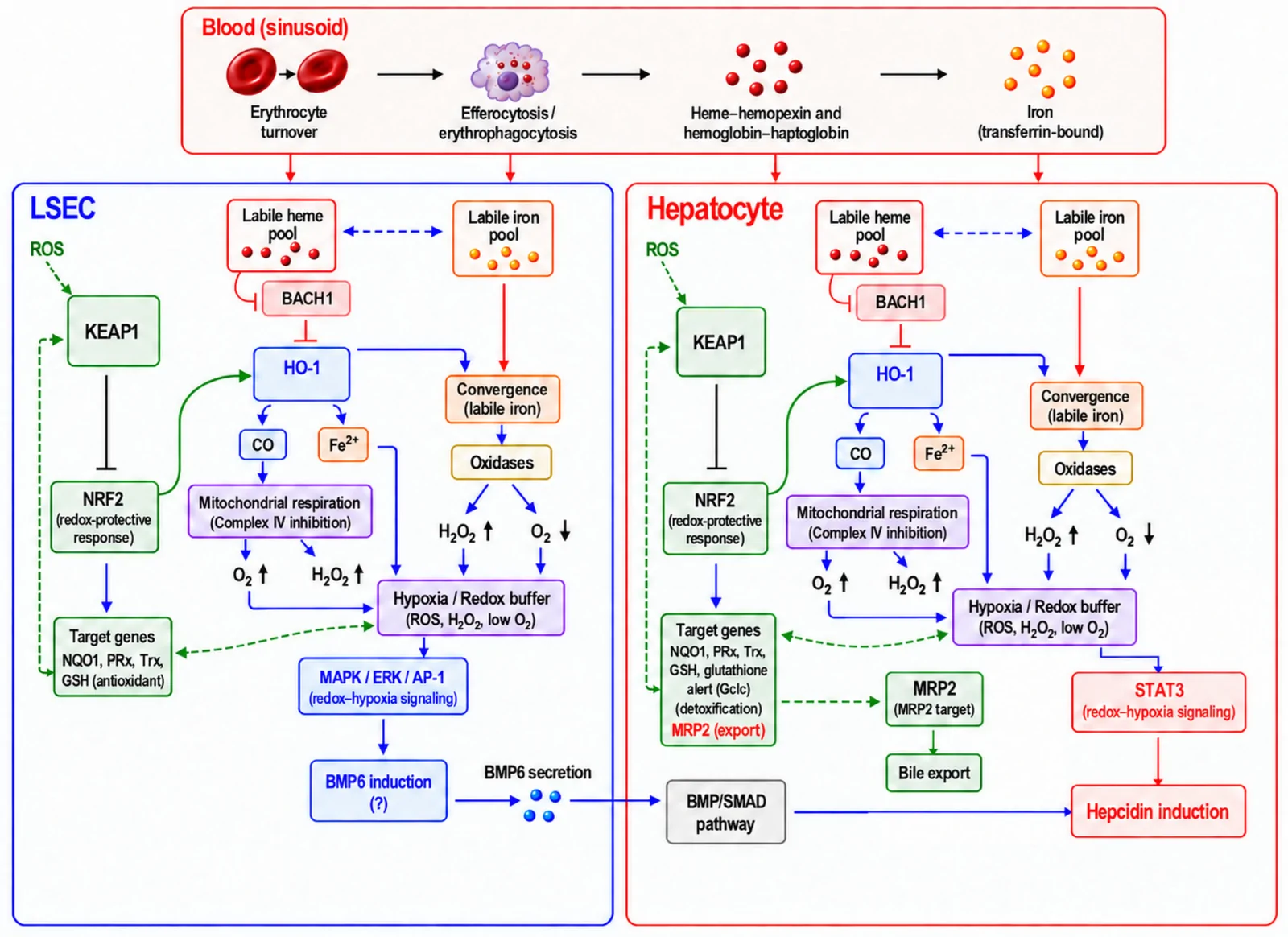
Proposed heme–iron–redox sensing network linking erythrocyte turnover to endothelial BMP6 and hepatocellular hepcidin regulation. Erythrocyte turnover supplies heme and iron to LSECs and hepatocytes, expanding the labile heme and iron pools. Heme binds to BACH1 and promotes its proteasomal degradation, thereby derepressing HO-1, which degrades heme to biliverdin, CO, and Fe^2+^. BACH1 thus functions as a specific intracellular heme sensor, whereas the KEAP1–NRF2 system acts as a general redox-sensing and cytoprotective pathway rather than as a specific heme sensor. Heme- and iron-derived redox signals modulate KEAP1–NRF2-dependent cytoprotection and H_2_O_2_/O_2_-sensitive redox buffering. In LSECs, these signals are proposed to promote MAPK/ERK/AP-1-dependent BMP6 induction and secretion, followed by hepatocellular BMP/SMAD signaling. In hepatocytes, oxygen–redox signaling converges on STAT3 and contributes to hepcidin induction, while NRF2 supports MRP2 expression and biliary export capacity. This link is particularly noteworthy because NRF2-dependent MRP2 expression connects erythrocyte-derived heme and redox signaling not only to hepcidin regulation but also to the biliary excretion of bilirubin conjugates, thereby coupling hemolysis directly to hepatic bilirubin clearance. Figure 7C illustrates the biphasic response, with moderate heme/iron flux inducing BMP6 and hepcidin and excessive flux promoting suppression or dysregulation. Although the labile heme and iron pools are shown as interconnected components of this network, their direct interactions and reciprocal regulation remain largely unexplored. Intracellular iron loading additionally inactivates IRP-mediated translational repression, thereby inducing ferritin synthesis as a cell-autonomous iron-storage response in macrophages, hepatocytes, and other iron-exposed cells. Abbreviations: AP-1, activator protein 1; BACH1, BTB and CNC homology protein 1; BMP6, bone morphogenetic protein 6; CO, carbon monoxide; ERK, extracellular signal-regulated kinase; Fe^2+^, ferrous iron; H_2_O_2_, hydrogen peroxide; HO-1, heme oxygenase-1; KEAP1, Kelch-like ECH-associated protein 1; LSEC, liver sinusoidal endothelial cell; MAPK, mitogen-activated protein kinase; MRP2, multidrug resistance-associated protein 2; NRF2, nuclear factor erythroid 2-related factor 2; ROS, reactive oxygen species; SMAD, mothers against decapentaplegic homolog; STAT3, signal transducer and activator of transcription 3.

Evidence for such upstream sensing comes from endothelial–hepatocellular coculture studies showing that ferric iron and low-nanomolar hemin induce endothelial BMP6 secretion, hepatocellular SMAD activation, and hepcidin expression [87]. These observations indicate that endothelial cells can translate exposure to iron and heme into a BMP6 signal but do not identify a single molecular iron or heme sensor. Instead, hepatic sensing may depend on an integrated multicellular network in which LSECs and hepatocytes monitor erythrocyte-derived heme, iron, and the accompanying redox changes in parallel (Figure 9).

In both cell types, the labile heme and iron pools provide a potential interface between erythrocyte-derived material and cellular signaling. Uptake of erythrocytes, hemoglobin, heme complexes, or extracellular heme can expand the labile heme pool, which dynamically controls heme allocation into hemoproteins [109]. HO-1-mediated degradation of heme generates biliverdin, CO, and Fe^2+^, thereby transferring material from the labile heme pool into the labile iron pool while simultaneously producing a regulator of mitochondrial respiration [78]. The quantitative dynamics and reciprocal regulation of these pools remain largely unexplored, but their direct metabolic connection makes them plausible components of a cellular system that senses erythrocyte-derived material as a flux rather than iron as an isolated signal.

Heme allocation additionally connects these pools to NO signaling because the induction and activity of the heme-containing enzyme iNOS depend on intracellular labile heme availability [109]. Heme-derived CO and NOS-derived NO can inhibit cytochrome c oxidase and thereby alter mitochondrial oxygen consumption, intracellular oxygen distribution, oxidase activity, and ROS formation [110]. Consequently, changes in erythrocyte-derived heme may be converted not only into changes in intracellular iron but also into oxygen- and redox-dependent signals capable of influencing BMP6 and hepcidin regulation.

BACH1 and KEAP1–NRF2 provide two complementary molecular interfaces within this network. Heme binding promotes proteasomal degradation of BACH1 and relieves BACH1-mediated repression of HO-1, whereas H_2_O_2_-dependent oxidation of KEAP1 stabilizes NRF2 and induces cytoprotective and iron-handling pathways [111,112,113,114]. The BACH1 pathway can establish distinct heme-metabolizing, iron-rich cellular programs characterized by coordinated changes in HO-1, ferritin, ferroportin, and heme transport [115]. NRF2 additionally links redox sensing to hepatobiliary handling of heme-derived products, including MRP2-mediated bilirubin export. Glutathione, thioredoxin, and peroxiredoxins further determine the magnitude and duration of the resulting redox signals [49]. Thus, BACH1 responds primarily to heme availability, whereas KEAP1–NRF2 responds to the associated redox environment; together they may translate changes in the labile heme and iron pools into coordinated cellular responses.

This concept is particularly relevant to the unresolved regulation of endothelial BMP6. Ferric iron and low-nanomolar hemin induce endothelial BMP6 secretion, while additional studies implicate NTBI–oxidative stress–NRF2 signaling and MAPK-dependent responses to heme, iron, H_2_O_2_, myoglobin, and inflammatory TLR ligands in LSECs [87,116,117]. JunB, c-Jun/AP-1-related signaling, and p38/JNK–ETS1 pathways have also been implicated in BMP6 regulation, although direct promoter binding and the hierarchy of these pathways remain incompletely established [118,119,120]. BMP6 should therefore be regarded as the endothelial output of upstream heme–iron–redox and inflammatory signal integration rather than as the primary iron sensor itself.

In parallel, hepatocytes take up erythrocyte-derived heme and iron through soluble uptake pathways and direct uptake of senescent or damaged erythrocytes [3,4,5]. They therefore possess their own intracellular heme–iron–redox sensing system while simultaneously receiving endothelial BMP6. The BMP6–BMP receptor–SMAD pathway constitutes the canonical extracellular input, whereas the redox-sensitive STAT3 response element of the hepcidin promoter provides a second point of integration. Besides cytokine-dependent JAK activation, STAT3 is modulated by sustained low-level H_2_O_2_, NOX4, physiological oxygen tension, and peroxiredoxin-dependent redox relays [43,45,46,51]. Sustained physiological H_2_O_2_ signaling differs fundamentally from bolus oxidative stress and may therefore convey information about continuous heme and iron flux rather than nonspecific cellular injury [45,48,50].

The model shown in Figure 9 therefore replaces the search for a single hepatic iron sensor with a distributed and cooperative sensing architecture. LSECs and hepatocytes both process erythrocyte-derived heme and iron through interconnected labile heme and iron pools, BACH1–HO-1 and KEAP1–NRF2 signaling, gasotransmitter-dependent mitochondrial modulation, and cellular redox pathways. LSECs translate these inputs into BMP6, whereas hepatocytes combine the resulting BMP–SMAD signal with their own intracellular heme–iron metabolism and redox-sensitive STAT3 signaling at the hepcidin promoter. Hepcidin thus emerges as the integrated output of two coupled cellular sensing systems that jointly monitor continuous erythrocyte flux, with the balance between their inductive and suppressive signals providing a mechanistic basis for the biphasic hepcidin response as this flux increases.

## 14. The Red Blood Cell–Liver Axis: Integrating Erythrocyte-Derived Signals with Hepatic Metabolism and Therapeutic Opportunities

For more than a century, the liver has primarily been viewed as the body’s central metabolic organ, whereas red blood cell biology, iron metabolism, and bilirubin metabolism have largely been studied as separate fields. Classical descriptions emphasize hepatic carbohydrate, lipid, and protein metabolism, detoxification, bile production, and nutrient storage. These functions remain fundamental, but the observations discussed throughout this article suggest that they may be integrated with another central biological task: the continuous processing of erythrocyte-derived material.

Within this framework, physiological erythrocyte turnover, eryptosis, erythrophagocytosis, efferocytosis, and intravascular hemolysis represent different positions along a continuous spectrum of erythrocyte injury and clearance. Under physiological conditions, controlled removal permits efficient recycling of hemoglobin, heme, and iron while limiting extracellular toxicity. Oxidative stress, inflammation, alcohol exposure, infection, mechanical injury, ineffective erythropoiesis, and advanced liver disease can shift this balance toward accelerated erythrocyte destruction and increased hepatic exposure to erythrocyte-derived substrates [3,24,86].

Hemoglobin, heme, and iron should not be regarded as interchangeable signals. Hemoglobin is processed through specialized scavenger pathways; heme is a bioactive and potentially cytotoxic molecule that activates inflammatory, redox, and cytoprotective responses; and iron is both an essential nutrient and a catalyst of oxidative reactions. The liver integrates these signals according to their molecular form, route of entry, concentration, cellular target, and physiological context. This distinction helps explain why erythrocyte uptake, hemolysis, heme, iron salts, inflammation, hypoxia, and erythropoietic demand can produce divergent effects on hepcidin regulation [9,87,88,121,122].

The RBC–liver axis also connects erythrocyte turnover to broader hepatic functions. Processing of erythrocyte-derived membrane lipids, globin, heme, iron, and bilirubin precursors intersects with protein trafficking, carrier-protein availability, lipid and cholesterol metabolism, redox defense, conjugation reactions, and biliary excretion. Albumin binds unconjugated bilirubin, fatty acids, and other hydrophobic metabolites, whereas transferrin coordinates systemic iron transport [92,123]. Haptoglobin and hemopexin limit the toxicity of extracellular hemoglobin and heme, while CD163- and CD91/LRP1-mediated pathways facilitate their clearance [83,84]. Heme degradation by HO-1 generates biliverdin, bilirubin, carbon monoxide, and iron, while bilirubin is conjugated by UGT1A1 and exported through canalicular transporters including MRP2 [29,30,31,78]. Heme-responsive NRF2 signaling may further connect erythrocyte-derived redox signals with MRP2 expression and biliary bilirubin export [124,125]. These links suggest that protein handling, lipid metabolism, bile formation, and detoxification may represent partially interconnected responses to the continuous hepatic processing of erythrocyte-derived material. At present, however, this broader interpretation remains a systems-level hypothesis requiring further validation.

The same framework may help explain clinical associations that otherwise appear unrelated. Disturbed erythrocyte turnover in alcohol-associated liver disease and advanced cirrhosis is associated with altered hepcidin regulation, iron accumulation, macrophage activation, impaired carrier-protein availability, cholestasis, fibrosis, and adverse outcome [3,24]. The combined pattern of erythrocyte count, macrocytosis, bilirubin, LDH, ferritin, sCD163, albumin, transferrin, haptoglobin, and hemopexin may therefore provide an integrated readout of erythrocyte processing, heme handling, iron redistribution, and hepatic dysfunction [24,83,84,92,123]. The classical view and the proposed RBC–liver axis framework are summarized in Table 1, whereas the supporting evidence, current evidence status, and remaining uncertainties are summarized in Table 2.

This expanded view also has developmental and evolutionary plausibility. During mammalian embryogenesis, the fetal liver is the principal hematopoietic organ before blood formation shifts to the bone marrow [17,18]. Hepcidin-like peptides combine antimicrobial and iron-regulatory functions across diverse animal lineages, suggesting that host defense and iron regulation co-evolved before their integration into the vertebrate hepcidin–ferroportin system [21,22]. The adult liver may therefore retain elements of an ancient biological program linking hematopoiesis, erythrocyte clearance, heme metabolism, iron regulation, antimicrobial defense, and the safe elimination of hydrophobic and redox-active products.

The RBC–liver axis also suggests several potential therapeutic opportunities. First, stabilization of erythrocytes could interrupt the reciprocal cycle in which eryptosis, hemolysis, and erythrophagocytosis increase hepatic exposure to hemoglobin, heme, iron, and bilirubin precursors, while hepatic dysfunction and cholestasis further destabilize erythrocytes [3,4,9,86]. N-acetylcysteine is of interest because it inhibits ethanol-induced erythrocyte injury and hepatic erythrophagocytosis experimentally, although its clinical effects remain context-dependent [3,126]. Redox-directed treatment should not, however, suppress physiological redox signaling indiscriminately, because sustained low-level H_2_O_2_ can contribute to STAT3-dependent hepcidin activation, whereas excessive or dysregulated oxidative stress may suppress hepcidin and promote injury [45,127].

Second, toxic erythrocyte-derived products could be intercepted. Haptoglobin binds cell-free hemoglobin and promotes CD163-dependent clearance, whereas hemopexin captures heme for uptake through CD91/LRP1 [83,84]. Hemopexin replacement remains experimental but has shown protective effects against heme-mediated injury in preclinical models [128]. Iron chelation may additionally limit labile iron and oxidative injury in selected settings, although its role in alcohol-associated liver disease, acute-on-chronic liver failure, and accelerated RBC turnover remains undefined [92,129]. Preservation or restoration of hepatobiliary transport, including MRP2-, BSEP-, and MDR3-dependent pathways, may represent another potential strategy for interrupting the feedback between cholestasis, erythrocyte injury, and hepatic heme exposure [30,31,124,130].

Finally, the hepcidin–ferroportin system may require disease- and phase-specific modulation. Hepcidin agonism could be useful during a hepcidin-suppressed phase of the proposed biphasic response or in genetic hepcidin deficiency, whereas excessive hepcidin activity may require the opposite approach [42,88,92]. Minihepcidins can reduce iron overload experimentally [131], but fixed agonism could aggravate iron-restricted anemia during ongoing hemolysis or increased erythropoietic demand. Therapeutic interventions should therefore target the dominant component of the disease process—RBC destabilization, extracellular hemoglobin, heme, labile iron, hepatobiliary retention, or maladaptive hepcidin regulation—rather than relying on nonspecific antioxidant treatment or indiscriminate manipulation of iron metabolism.

The RBC–liver axis should therefore be regarded as a working framework rather than a final explanation. Its central hypothesis is that the liver continuously senses and processes signals generated by lifelong erythrocyte turnover and translates them into metabolic, immunological, redox, biliary, and systemic regulatory responses. The liver is consequently not merely a metabolic organ receiving the end products of erythrocyte degradation, but a central sensor, processor, and regulator of erythrocyte biology.

**Table 2:** Evidence map of the RBC–Liver Axis: Established findings, preliminary evidence, and remaining questions. The table summarizes the principal components of the proposed RBC–liver axis and distinguishes evidence status from remaining uncertainty. Evidence categories indicate established physiology, published experimental evidence, human disease evidence, preliminary unpublished findings requiring validation, mechanistically plausible pathways whose integration remains unproven, and systems-level hypotheses requiring further testing.

Component of the RBC–Liver Axis	Supporting Evidence and References	Current Evidence Status	What Remains to be Established
Daily RBC turnover provides approximately 20–25 mg iron/day	Physiological RBC lifespan and iron recycling; daily RBC-derived iron flux exceeds intestinal absorption [2,92].	Established physiology	Quantitative distribution of this flux among spleen, Kupffer cells, LSECs, hepatocytes, and circulating scavenger systems.
RBC damage, eryptosis, phosphatidylserine exposure, and erythrocyte clearance	Oxidative and metabolic stressors, including heme, bilirubin, and bile acids, promote RBC membrane damage, eryptosis, and phosphatidylserine exposure, thereby facilitating erythrophagocytosis and efferocytosis. NAC attenuates this process by reducing oxidative stress [3,4,132,133]	Supported by published experimental and disease-related evidence	Relative contribution of individual stressors and their quantitative impact on RBC clearance in vivo.
Macrophage-mediated erythrophagocytosis and iron recycling	Extensive physiological and experimental literature on erythrophagocytosis, ferroportin-dependent iron export, and recycling to erythropoiesis [26,28,88].	Established	Relative contribution of splenic versus hepatic compartments under basal and pathological conditions.
Hepatic uptake of damaged RBCs by LSECs	Published experimental evidence for LSEC uptake and processing of damaged RBCs [4,5].	Supported by published experimental evidence	Direct quantitative in vivo contribution, uptake kinetics, and downstream intracellular processing.
Hepatocyte uptake of damaged RBCs	Published experimental evidence for direct hepatocyte uptake of damaged or senescent RBCs [5], complemented by suggestive human histological evidence of hepatocellular RBC [103] or RBC-derived material [3,4].	Supported by published experimental evidence	Distinction between direct RBC uptake and intracellular RBC-derived hemoglobin or iron; quantitative in vivo contribution and relative importance compared with macrophages and LSECs.
RBC-derived material reaches the human liver in ALD	Human liver tissue studies demonstrate hemoglobin accumulation in macrophages, LSECs, and hepatocytes in association with hemolysis [3,4].	Supported in human disease	Generalizability across etiologies, disease stages, and degrees of hemolysis.
Physiological RBC turnover may contribute to basal hepcidin regulation	Established daily RBC turnover and hepcidin biology provide the physiological rationale. Preliminary data from our laboratory suggest that RBC-derived heme and iron can stimulate hepatocyte hepcidin expression (manuscript in preparation).	Preliminary; unpublished; requires independent validation	Direct demonstration that physiological RBC-derived flux contributes to the basal hepcidin set point.
Biphasic hepcidin response to increasing heme/iron flux	The biphasic hepcidin concept was first proposed in [9], based on stimulatory effects of low heme concentrations in hepatocytes and macrophages and suppressive effects of higher heme or iron concentrations. Preliminary phenylhydrazine-induced hemolysis data from our laboratory extend this model in vivo (manuscript in preparation, [87])	Published conceptual framework; preliminary experimental support; requires further validation	The precise relationship between HFE-dependent sensing, endothelial BMP6–SMAD signaling, and H_2_O_2_/STAT3 activation in vivo, including the relative contribution of these pathways to the stimulatory and suppressive phases.
H_2_O_2_-mediated hepcidin stimulation and hypoxic amplification	In vitro studies show that sustained submicromolar H_2_O_2_ stimulates hepcidin expression through redox-sensitive STAT3 signaling, and that hypoxia enhances this response. H_2_O_2_ may arise from mitochondrial respiration, peroxisomes, NOX enzymes, and other oxidases [45,46]	Supported by published experimental evidence; integrated role in the RBC–liver axis requires further validation	In vivo validation of the H_2_O_2_- and hypoxia-dependent hepcidin response, including the relative contribution of different H_2_O_2_ sources and interaction with BMP6–SMAD signaling.
Heme–CO/NO–O_2_–H_2_O_2_–STAT3 coupling	Individual components are supported by studies of HO-1, gasotransmitters, mitochondrial respiration, H_2_O_2_, oxygen tension, and STAT3 [45,46,51,78].	Individual components supported; integrated pathway inferred	Validation of the complete pathway as a connected, flux-dependent mechanism in vivo.
RBC turnover contributes to ALD iron loading and clinical outcome	Human associations between hemolysis/RBC turnover, hepatic iron, macrophage activation, fibrosis, and mortality [3,6].	Supported association; causality requires further study	Prospective and interventional evidence that altered RBC turnover causally contributes to iron loading, fibrosis, and outcome.
RBC processing influences broader protein, lipid, bile, and gene-regulatory functions	Systems-level integration of erythrocyte-derived heme, iron, bilirubin, membrane lipids, redox signals, carrier proteins, MRP2, NRF2, FXR, PXR, HNF4α, and LXR [30,31,123,125,134].	Systems-level hypothesis	Direct mechanistic and quantitative testing of the proposed links beyond heme, iron, bilirubin, and hepcidin biology.

## 15. Conclusions

In conclusion, the red blood cell–liver axis provides a unifying framework linking erythrocyte turnover to hepatic iron sensing, heme metabolism, redox signaling, lipid handling, bile formation, and chronic liver disease. Daily erythrocyte turnover generates the dominant physiological iron flux in the body, suggesting that the liver continuously senses RBC-derived material rather than body iron stores alone. Recent findings showing erythrocyte uptake by hepatocytes and liver sinusoidal endothelial cells expand erythrocyte clearance beyond classical macrophage biology. Moreover, erythrocyte and hemoglobin uptake may reprogram Kupffer cells, and potentially LSECs and hepatocytes, toward specialized HO-1/NRF2-dependent, iron-recycling, and inflammation-limiting states, suggesting that RBC-derived material regulates hepatic cell identity as well as metabolism. Within this framework, hepcidin emerges as an integrated output of heme and iron flux, inflammation, oxygen tension, redox status, and erythropoietic demand. A biphasic model is proposed in which physiological erythrocyte-derived heme and iron stimulate hepcidin, whereas excessive hemolysis or heme overload may suppress or dysregulate this response. Redox-sensitive H_2_O_2_–STAT3 signaling, amplified by hypoxia and oxidases such as NOX4, may provide a mechanistic link between erythrocyte-derived signals and hepcidin regulation. Alcohol-associated liver disease illustrates the clinical relevance of this axis, as erythrocyte fragility, hemolysis, erythrophagocytosis, iron accumulation, fibrosis, and mortality converge. Beyond ALD, altered erythrocyte turnover may represent a common and previously underrecognized pathogenic mechanism across diverse liver diseases, including NAFLD/MASLD and Wilson disease, and become an increasingly self-amplifying driver as liver disease advances. By coupling the hepatic delivery of redox-active heme and iron with the processing of erythrocyte membrane lipids, increased RBC turnover may also promote lipid peroxidation and ferroptotic liver injury.

This framework also changes how the liver itself can be understood. Rather than being merely the body’s largest metabolic organ, it may represent the central organ that senses and processes the lifelong erythrocyte flux, ranging from intact cells and hemolysis-derived molecules to heme degradation, iron recycling, membrane lipid and cholesterol handling, and biliary bilirubin excretion. Seen in this light, albumin production, lipid metabolism, redox defense, detoxification, and bile formation are not simply parallel hepatic functions. They may be interconnected adaptations that allow the liver to transport, neutralize, recycle, and ultimately eliminate erythrocyte-derived hydrophobic and redox-active products. Thus, the continuous processing of erythrocytes may be one of the fundamental biological tasks that shaped the evolution and function of the liver.

## Figures and Tables

**Table 1:** Conceptual limitations of the classical spleen-centered view and the proposed RBC–liver axis. The table contrasts conventional views of erythrocyte degradation, bilirubin handling, hepcidin regulation, and hepatic iron overload with the RBC–liver axis framework. This concept proposes that Kupffer cells, liver sinusoidal endothelial cells, and hepatocytes form a hepatic network that continuously processes erythrocyte-derived hemoglobin, heme, iron, membrane lipids, bilirubin precursors, and redox-active signals. Abbreviations: ALD, alcohol-associated liver disease; BMP6, bone morphogenetic protein 6; HFE, homeostatic iron regulator; LDH, lactate dehydrogenase; LSEC, liver sinusoidal endothelial cell; MCV, mean corpuscular volume; NAC, N-acetylcysteine; PCLS, precision-cut liver slices; RBC, red blood cell; sCD163, soluble CD163; SMAD, mothers against decapentaplegic homolog.

Classical View	RBC–Liver Axis Interpretation	Key Supporting Observations
RBC recycling is centered on the spleen and reticuloendothelial macrophages; hepatic handling is secondary.	Kupffer cells, LSECs, and hepatocytes form a multicellular hepatic network that continuously processes damaged RBCs and their hemoglobin, heme, iron, lipids, and bilirubin precursors.	Damaged RBCs are also taken up by hepatocytes, LSECs, and hepatic macrophages in experimental models [3,4,5].
The liver mainly excretes bilirubin.	Bilirubin is one output of integrated hepatic heme degradation, iron recycling, bile formation, and redox signaling.	The RBC–liver axis links bilirubin formation to heme degradation, iron handling, and redox-active signaling.
ALD is attributed mainly to steatosis, inflammation, fibrosis, and cirrhosis.	Hemolysis supplies hepatic hemoglobin, heme, lipids, and iron and may promote oxidative stress, iron accumulation, fibrosis, and mortality in ALD.	Serum hemolysis occurs in ALD and is reversible with abstinence; the alcohol → hemolysis → iron overload → fibrosis cascade is experimentally supported [3].
Ethanol toxicity is interpreted mainly as direct hepatocellular or inflammatory injury.	Ethanol also induces eryptosis and primes damaged RBCs for hepatic erythrophagocytosis, increasing heme and iron exposure.	Alcoholic RBCs show increased stress fragility; ethanol induces eryptosis and enhances erythrophagocytosis [3].
sCD163 and hepatic macrophage activation are regarded as nonspecific markers of inflammation.	sCD163 reflects the hemoglobin–haptoglobin clearance, while CD163-positive hepatic macrophages directly process RBC-derived material and link hemolysis to fibrogenic signaling.	Hepatic CD163+ macrophages ingest RBCs in vivo; sCD163 tracks hemolysis/erythrophagocytosis; hepatic CD163 upregulation is associated with fibrosis; damaged RBCs induce CD163 in PCLS [3].
Hepcidin mainly reflects body iron stores.	Hepcidin integrates RBC-derived heme and iron flux with BMP6–SMAD signaling, redox status, oxygen tension, inflammation, and erythropoietic demand.	Heme and iron produce a biphasic hepcidin response: HFE-dependent induction after mild hemolysis and suppression during severe hemolysis (in preparation).
Heme and bilirubin are viewed mainly as degradation products or toxicity markers; antioxidant effects are considered nonspecific hepatoprotection.	Heme and bilirubin can amplify erythrophagocytosis, while redox intervention can interrupt ethanol-induced RBC injury and hepatic uptake.	Heme and bilirubin enhance erythrophagocytosis experimentally; NAC blocks ethanol-induced erythrophagocytosis [3].
Hepatic hemoglobin accumulation is difficult to identify, and erythrophagocytosis is commonly studied in isolated macrophage systems.	Hemoglobin imaging, human erythrophagocytosis assays, and liver tissue models permit direct assessment of hepatic RBC uptake and heme processing across cell types.	Hemoglobin autofluorescence and immunofluorescence identify hepatic RBC-derived material; human in vitro and PCLS models measure RBC uptake and CD163 induction [3,4].
Albumin, transferrin, haptoglobin, and hemopexin are treated as separate synthetic or scavenger markers.	Together, these proteins reflect carrier availability and hepatic handling of bilirubin, heme, iron, fatty acids, and other RBC-derived metabolites.	Their inverse associations with sCD163, bilirubin, LDH, MCV, ferritin, and liver stiffness support an integrated RBC–liver phenotype [9].
Hepatic iron overload is explained mainly by intestinal absorption, iron stores, inflammation, or genetics.	Recycled erythrocyte iron is the dominant physiological iron flux reaching the liver and may become pathogenic when RBC destruction increases.	Approximately 20–25 mg of RBC iron is recycled daily versus 1–2 mg absorbed intestinally; hemolysis promotes iron overload and fibrosis.
The liver is primarily regarded as a metabolic, detoxifying, and biliary organ.	The liver is also a central sensor, processor, and regulator of RBC-derived material, linking erythrocyte turnover to heme metabolism, iron homeostasis, bile formation, protein handling, fibrosis, and mortality.	The unified RBC–liver axis integrates RBC turnover, heme and iron sensing, bilirubin and bile formation, carrier proteins, fibrogenesis, and clinical outcome.

## Data Availability

No new data were created or analyzed in this study. Data sharing is not applicable to this article.

## Supplementary Materials

The following supporting information can be downloaded at: https://www.mdpi.com/article/10.3390/biology15191773/s1, Supplemental Table S1. Macrocytosis identifies a hemolysis-linked RBC–liver phenotype associated with increased mortality in heavy drinkers. Heavy drinkers were stratified by mean corpuscular volume (MCV) into high-MCV (>96 fL), normal-MCV (80–96 fL), and low-MCV (<80 fL) groups. Compared with the normal-MCV group, the high-MCV group had higher soluble CD163 (sCD163) levels, greater proportions of elevated sCD163 and ferritin values, a higher prevalence of anemia, alterations in erythropoietic and iron-regulatory parameters, and markedly higher mortality. Notably, hepcidin levels were elevated across all MCV groups relative to healthy controls, consistent with findings from murine models of chronic alcohol exposure. *p* values refer to comparisons between the high-MCV and normal-MCV groups: * *p* < 0.05, ** *p* < 0.01, *** *p* < 0.005. Data from *n* = 1087 patients. Supplemental Figure S1. Hepatic iron accumulation during (A) progression of alcohol-related liver disease (ALD), the most common liver disease worldwide, and typical histological staining or pathological iron deposits by Prussian blue in (B) ALD and (C) hereditary hemochromatosis. (A) Schematic illustration of progressive hepatic iron accumulation in ALD during the transition from healthy liver to steatosis, steatohepatitis, cirrhosis, and HCC, with a slight decline in advanced HCC reflecting tumor-associated metabolic reprogramming and replacement of normal hepatocytes. (B) Representative Perls’ Prussian blue staining from a patient with ALD showing steatosis, advanced fibrosis, and mild mixed iron deposition involving both hepatocytes and Kupffer cells/macrophages. This pattern is consistent with increased erythrocyte turnover, erythrophagocytosis, low-grade hemolysis, and hepatic exposure to heme-derived iron. (C) Representative Perls’ Prussian blue staining from a patient with HFE-associated hereditary hemochromatosis showing marked hepatocellular iron overload with relative centrilobular sparing around the central vein (CV), consistent with the typical periportal pattern of HFE-related iron accumulation. Abbreviations: ALD, alcohol-associated liver disease; CV, central vein; HCC, hepatocellular carcinoma; HFE, homeostatic iron regulator gene. Supplemental Figure S2. Major signaling pathways controlling hepatocellular hepcidin expression. Canonical BMP2/6–SMAD signaling is regulated by transferrin-bound iron sensing through TfR1/TfR2 and HFE and by the co-receptors HJV and neogenin, whereas TMPRSS6 and SMAD7 provide inhibitory control. ERFE acts as a major erythroid suppressor of hepcidin through inhibition of BMP–SMAD signaling, although it does not fully account for hepcidin suppression under all conditions. Inflammatory cytokines activate the JAK–STAT3 pathway. H_2_O_2_ generated by NADPH oxidases and DUOX modulates STAT3 activity and interacts with physiological oxygen tension and cellular redox buffering. This redox-sensitive response is inhibited by NAC and may be further influenced by HO-1-derived CO through modulation of intracellular oxygen utilization and redox status. Hepcidin subsequently promotes ferroportin internalization in enterocytes, macrophages, and hepatocytes, thereby reducing intestinal iron absorption, cellular iron release, and plasma iron availability. Abbreviations: ACVR2A, activin A receptor type 2A; ALK2/3, activin receptor-like kinase 2/3; BMP, bone morphogenetic protein; BMP-RE, BMP-responsive element; BMPR, BMP receptor; CO, carbon monoxide; DUOX, dual oxidase; EPO, erythropoietin; ER, endoplasmic reticulum; ERFE, erythroferrone; FOXO1, forkhead box O1; HAMP, hepcidin antimicrobial peptide; HFE, homeostatic iron regulator; HJV, hemojuvelin; HO-1, heme oxygenase-1; IL, interleukin; JAK, Janus kinase; NAC, N-acetylcysteine; NOX, NADPH oxidase; NTBI, non-transferrin-bound iron; SMAD, mothers against decapentaplegic homolog; SMURF1, SMAD-specific E3 ubiquitin protein ligase 1; STAT3, signal transducer and activator of transcription 3; STAT3-RE, STAT3-responsive element; TfR, transferrin receptor; TMPRSS6, transmembrane serine protease 6; TNF-α, tumor necrosis factor-α. Supplemental Figure S3. Red blood cell turnover as a central determinant of hepatic signaling and biphasic hepcidin regulation. Schematic model showing erythrocyte turnover as a major determinant of hepatic heme and iron sensing. Under physiological conditions, approximately 1% of circulating erythrocytes is processed daily, generating a constant flux of hemoglobin, heme, iron, membrane lipids, and redox-active molecules. These signals are sensed by sinusoidal endothelial cells, Kupffer cells, and hepatocytes. Hepatocytes integrate BMP6/SMAD signaling, transferrin receptor/HFE-mediated iron sensing, inflammatory cytokine signaling, and erythrocyte-derived redox pathways converging on STAT3. Moderate heme delivery stimulates hepcidin, whereas excessive heme or iron exposure causes oxidative overload and hepcidin suppression. Systemic modifiers, including EPO, ERFE, hypoxia, and IL-6, further shape the response. HFE loss-of-function may blunt the ascending physiological limb of the biphasic hepcidin response, whereas minihepcidins and targeted redox-, hemoglobin-, or heme-directed therapies, including haptoglobin and hemopexin replacement, could counteract its descending pathological limb by restoring hepcidin activity or limiting toxic overload. Supplemental Figure S4. Hemolysis and erythrophagocytosis increase with advancing liver fibrosis. Serum markers of erythrocyte turnover and macrophage-mediated erythrophagocytosis were analyzed according to histological fibrosis stage in 228 heavy drinkers. Shown are (A) soluble CD163, (B) hemoglobin, (C) total bilirubin, and (D) indirect bilirubin. Soluble CD163 increased progressively across histological fibrosis stages, from F0 (no fibrosis) to F4 (cirrhosis), indicating increasing hemoglobin–haptoglobin clearance and macrophage activation. In parallel, hemoglobin concentrations declined, whereas total and indirect bilirubin increased, particularly in advanced fibrosis and cirrhosis. The combined rise in sCD163 and indirect bilirubin together with falling hemoglobin supports the concept that disturbed erythrocyte turnover is an integral component of advanced chronic liver disease and the proposed RBC–liver axis. Data are shown as mean and 95% confidence interval. Asterisks indicate statistical significance versus F0 after adjustment for multiple comparisons using Dunnett’s method: * *p* < 0.05, ** *p* < 0.01, *** *p* < 0.001, **** *p* < 0.0001. Modified from [3]. F1 denotes portal fibrosis without septa, F2 portal fibrosis with few septa, and F3 numerous septa without cirrhosis.

## Abbreviations

ALD, alcohol-associated liver disease; ALT, alanine aminotransferase; AP-1, activator protein 1; ApoA1, apolipoprotein A-I; AST, aspartate aminotransferase; BACH1, BTB and CNC homology 1; BMP, bone morphogenetic protein; BMP6, bone morphogenetic protein 6; BSEP, bile salt export pump; CAT, catalase; CD163, cluster of differentiation 163; CD91/LRP1, cluster of differentiation 91/low-density lipoprotein receptor-related protein 1; CO, carbon monoxide; CRP, C-reactive protein; DAPI, 4′,6-diamidino-2-phenylindole; DUOX, dual oxidase; EPO, erythropoietin; ERFE, erythroferrone; ERK, extracellular signal-regulated kinase; ETS1, E26 transformation-specific transcription factor 1; FAC, ferric ammonium citrate; FPN, ferroportin; FXR, farnesoid X receptor; GGT, gamma-glutamyltransferase; GOX, glucose oxidase; GPX4, glutathione peroxidase 4; GSH, glutathione; H_2_O_2_, hydrogen peroxide; HAMP, hepcidin antimicrobial peptide; Hb, hemoglobin; HFE, homeostatic iron regulator; HIF-1α, hypoxia-inducible factor 1 alpha; HJV, hemojuvelin; HNF4α, hepatocyte nuclear factor 4 alpha; HO-1, heme oxygenase-1; IL, interleukin; iNOS, inducible nitric oxide synthase; IRE, iron-responsive element; IRP, iron regulatory protein; JAK, Janus kinase; JNK, c-Jun N-terminal kinase; KEAP1, Kelch-like ECH-associated protein 1; LDH, lactate dehydrogenase; LSEC, liver sinusoidal endothelial cell; LXR, liver X receptor; MAPK, mitogen-activated protein kinase; MASLD, metabolic dysfunction-associated steatotic liver disease; MCV, mean corpuscular volume; MDR3, multidrug resistance protein 3; MRP2, multidrug resistance-associated protein 2; NAC, N-acetylcysteine; NAFLD, non-alcoholic fatty liver disease; NASH, non-alcoholic steatohepatitis; NF-κB, nuclear factor kappa B; NO, nitric oxide; NOX, NADPH oxidase; NOX1, NADPH oxidase 1; NOX2, NADPH oxidase 2; NOX4, NADPH oxidase 4; NRF2, nuclear factor erythroid 2-related factor 2; NTBI, non-transferrin-bound iron; PCLS, precision-cut liver slices; PHZ, phenylhydrazine; PS, phosphatidylserine; PXR, pregnane X receptor; RBC, red blood cell; ROS, reactive oxygen species; sCD163, soluble cluster of differentiation 163; SMAD, mothers against decapentaplegic homolog; SOD, superoxide dismutase; STAT3, signal transducer and activator of transcription 3; TfR1, transferrin receptor 1; TLR, Toll-like receptor; TLR4, Toll-like receptor 4; Trx, thioredoxin; UGT1A1, UDP-glucuronosyltransferase 1A1; ZIP14, Zrt- and Irt-like protein 14.
