# Supplementary material for: The Red Blood Cell–Liver Axis: A New Framework for Iron Homeostasis and Liver Biology
Source: Biology (Basel). 2026 Oct 6;15(19):1773. doi: 10.3390/biology15191773 (PMC13664511; doi:10.3390/biology15191773)
Supplement: Supplementary file 1 [file biology-15-01773-s001.zip › biology-4501858-supplementary.pdf]

Supplemental Material

Supplemental Table S1. Macrocytosis identifies a hemolysis-linked RBC–liver phenotype associated with increased mortality in heavy drinkers.
Heavy drinkers were stratified by mean corpuscular volume (MCV) into high-MCV (>96 fL), normal-MCV (80–96 fL), and low-MCV (<80 fL) groups. Compared with the normal-MCV group, the high-MCV group had higher soluble CD163 (sCD163) levels, greater proportions of elevated sCD163 and ferritin values, a higher prevalence of anemia, alterations in erythropoietic and iron-regulatory parameters, and markedly higher mortality. Notably, hepcidin levels were elevated across all MCV groups relative to healthy controls, consistent with findings from murine models of chronic alcohol exposure. P values refer to comparisons between the high-MCV and normal-MCV groups: *P < 0.05, **P < 0.01, ***P < 0.005. Data from n=1087 patients.

Groups	Units	normal	elevated/ decreased	P*	All	high MCV >96	normal MCV 80-96	low MCV <80
**mean MCV**<br>**Percentage**	(fL)<br>(%)			***	93.4<br>100.0%	101.7<br>31.8%	90.4<br>65.7%	65.2<br>2.5%
**Hemoglobin**<br>**Anemia fraction**	(g/dL)		<12.5	***	14<br>19.7%	13.4<br>28.4%	14.4<br>13.0%	12.1<br>33.7%
**Ferritin**<br>**elevated Ferritin fraction**	(ng/mL)		>400	***	594<br>44.9%	853.4<br>62.9%	484.1<br>38.0%	272.1<br>20.0%
**CD163**<br>**elevated CD163 fraction**	(ng/mL)<br>(ng/mL)		>1500<br>>1500	***<br>***	1686.3<br>44.7%	1945.0<br>59.6%	1325.8<br>32.5%	1406.8<br>38.5%
**Vitamin B12**<br>**Folic acid**<br>**Erythropoietin**<br>**Hepcidin**	(pmol/L)<br>(nmol/L)<br>(mIU/mL)<br>(ng/mL)	145-596<br><br>6-15<br>1-55	 <7.1	 **<br>**<br>**	524.5<br>15.3<br>8.0<br>15.8	616.2<br>10.7<br>11.7<br>13.9	494.4<br>17.3<br>6.2<br>17.1	341.0<br>8.6<br>0.5<br>24.4
**Serum iron**	(ug/dl)	95-158			123.9	129.1	122.2	103.7
**Status death**				***		31.1%	11.6%	20.0%
P* comparison between high and normal MCV								

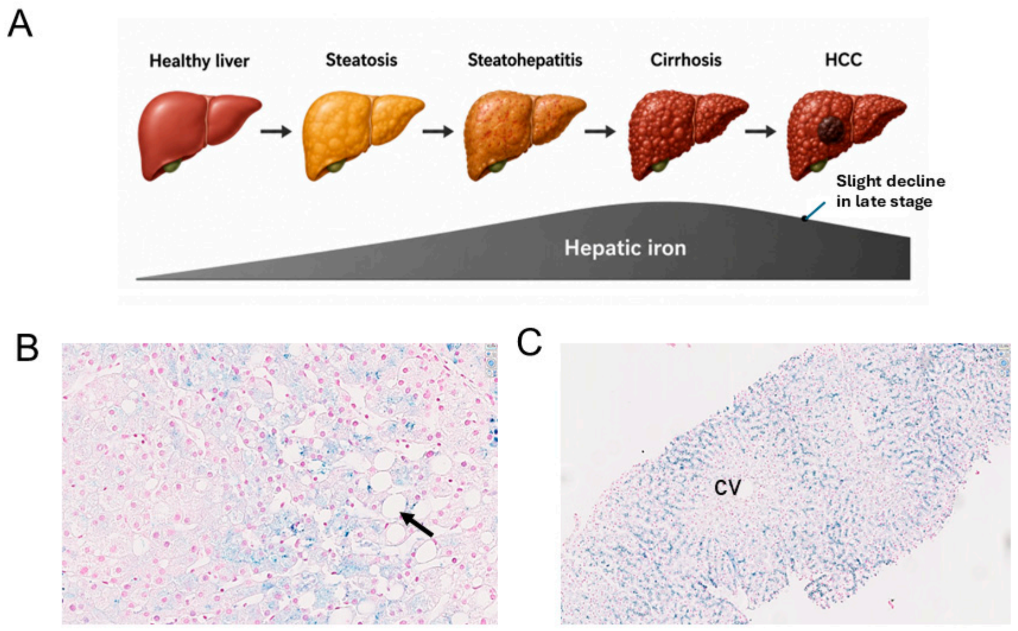

Suppl. Figure S1. Hepatic iron accumulation during A) progression of alcohol-related liver disease (ALD), the most common liver disease worldwide, and typical histological staining or pathological iron deposits by Prussian blue in B) ALD and C) hereditary hemochromatosis.

(A) Schematic illustration of progressive hepatic iron accumulation in ALD during the transition from healthy liver to steatosis, steatohepatitis, cirrhosis, and HCC, with a slight decline in advanced HCC reflecting tumor-associated metabolic reprogramming and replacement of normal hepatocytes.

(B) Representative Perls' Prussian blue staining from a patient with ALD showing steatosis, advanced fibrosis, and mild mixed iron deposition involving both hepatocytes and Kupffer cells/macrophages. This pattern is consistent with increased erythrocyte turnover, erythrophagocytosis, low-grade hemolysis, and hepatic exposure to heme-derived iron. (C) Representative Perls' Prussian blue staining from a patient with HFE-associated hereditary hemochromatosis showing marked hepatocellular iron overload with relative centrilobular sparing around the central vein (CV), consistent with the typical periportal pattern of HFE-related iron accumulation.

Abbreviations: ALD, alcohol-associated liver disease; CV, central vein; HCC, hepatocellular carcinoma; HFE, homeostatic iron regulator gene.

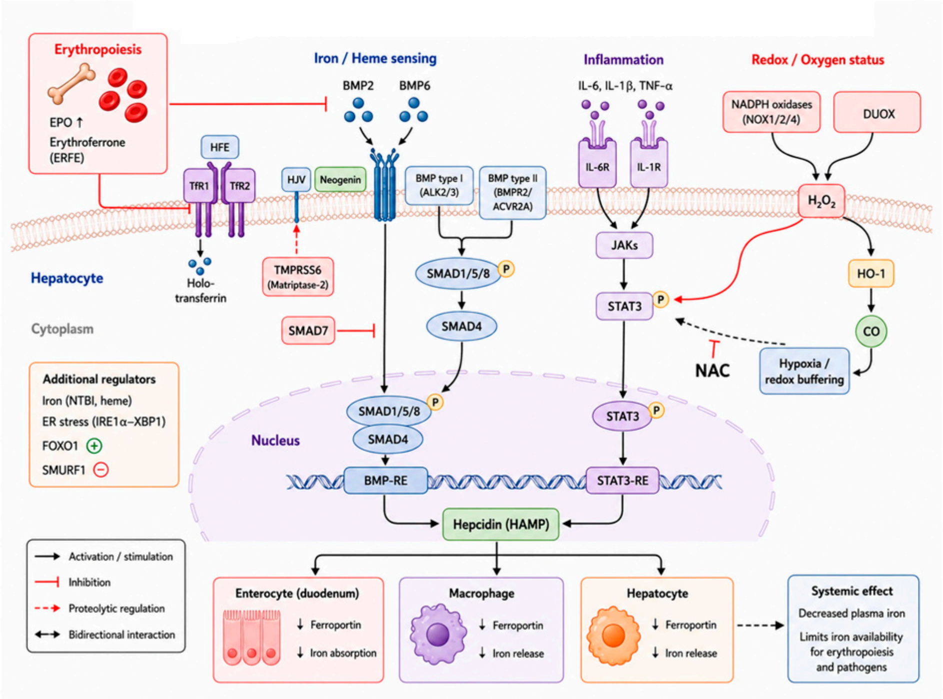

Suppl. Figure S2. Major signaling pathways controlling hepatocellular hepcidin expression. Canonical BMP2/6–SMAD signaling is regulated by transferrin-bound iron sensing through TfR1/TfR2 and HFE and by the co-receptors HJV and neogenin, whereas TMPRSS6 and SMAD7 provide inhibitory control. ERFE acts as a major erythroid suppressor of hepcidin through inhibition of BMP–SMAD signaling, although it does not fully account for hepcidin suppression under all conditions. Inflammatory cytokines activate the JAK–STAT3 pathway. H_2O_2 generated by NADPH oxidases and DUOX modulates STAT3 activity and interacts with physiological oxygen tension and cellular redox buffering. This redox-sensitive response is inhibited by NAC and may be further influenced by HO-1-derived CO through modulation of intracellular oxygen utilization and redox status. Hepcidin subsequently promotes ferroportin internalization in enterocytes, macrophages, and hepatocytes, thereby reducing intestinal iron absorption, cellular iron release, and plasma iron availability. Abbreviations: ACVR2A, activin A receptor type 2A; ALK2/3, activin receptor-like kinase 2/3; BMP, bone morphogenetic protein; BMP-RE, BMP-responsive element; BMPR, BMP receptor; CO, carbon monoxide; DUOX, dual oxidase; EPO, erythropoietin; ER, endoplasmic reticulum; ERFE, erythroferrone; FOXO1, forkhead box O1; HAMP, hepcidin antimicrobial peptide; HFE, homeostatic iron regulator; HJV, hemojuvelin; HO-1, heme oxygenase-1; IL, interleukin; JAK, Janus kinase; NAC, N-acetylcysteine; NOX, NADPH oxidase; NTBI, non-transferrin-bound iron; SMAD, mothers against decapentaplegic homolog; SMURF1, SMAD-specific E3 ubiquitin protein ligase 1; STAT3, signal transducer and activator of transcription 3; STAT3-RE, STAT3-responsive element; TfR, transferrin receptor; TMPRSS6, transmembrane serine protease 6; TNF-α, tumor necrosis factor-α.

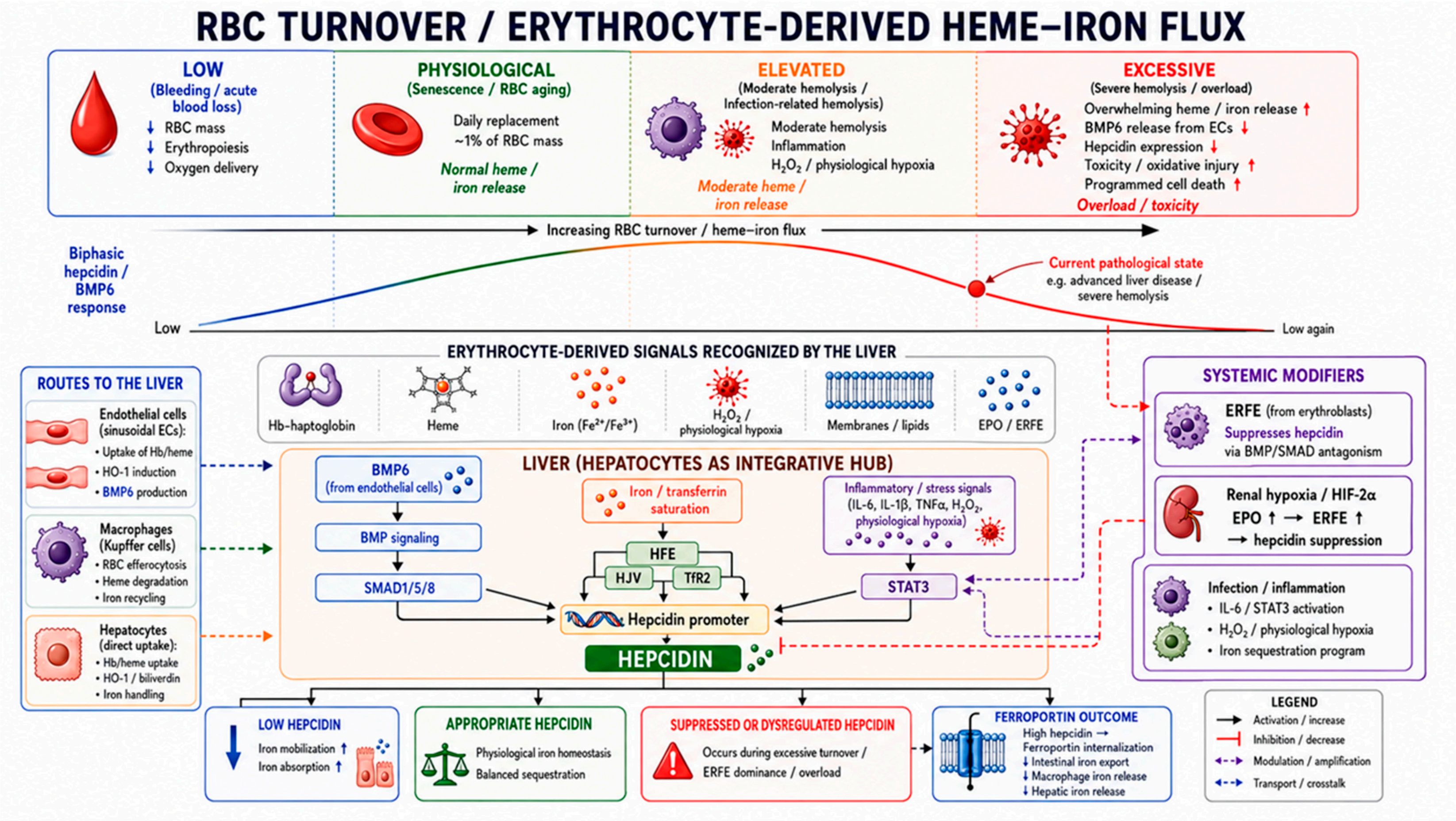

Suppl. Figure S3. Red blood cell turnover as a central determinant of hepatic signaling and biphasic hepcidin regulation

Schematic model showing erythrocyte turnover as a major determinant of hepatic heme and iron sensing. Under physiological conditions, approximately 1% of circulating erythrocytes is processed daily, generating a constant flux of hemoglobin, heme, iron, membrane lipids, and redox-active molecules. These signals are sensed by sinusoidal endothelial cells, Kupffer cells, and hepatocytes. Hepatocytes integrate BMP6/SMAD signaling, transferrin receptor/HFE-mediated iron sensing, inflammatory cytokine signaling, and erythrocyte-derived redox pathways converging on STAT3. Moderate heme delivery stimulates hepcidin, whereas excessive heme or iron exposure causes oxidative overload and hepcidin suppression. Systemic modifiers, including EPO, ERFE, hypoxia, and IL-6, further shape the response. HFE loss-of-function may blunt the ascending physiological limb of the biphasic hepcidin response, whereas minihepcidins and targeted redox-, hemoglobin-, or heme-directed therapies, including haptoglobin and hemopexin replacement, could counteract its descending pathological limb by restoring hepcidin activity or limiting toxic overload.

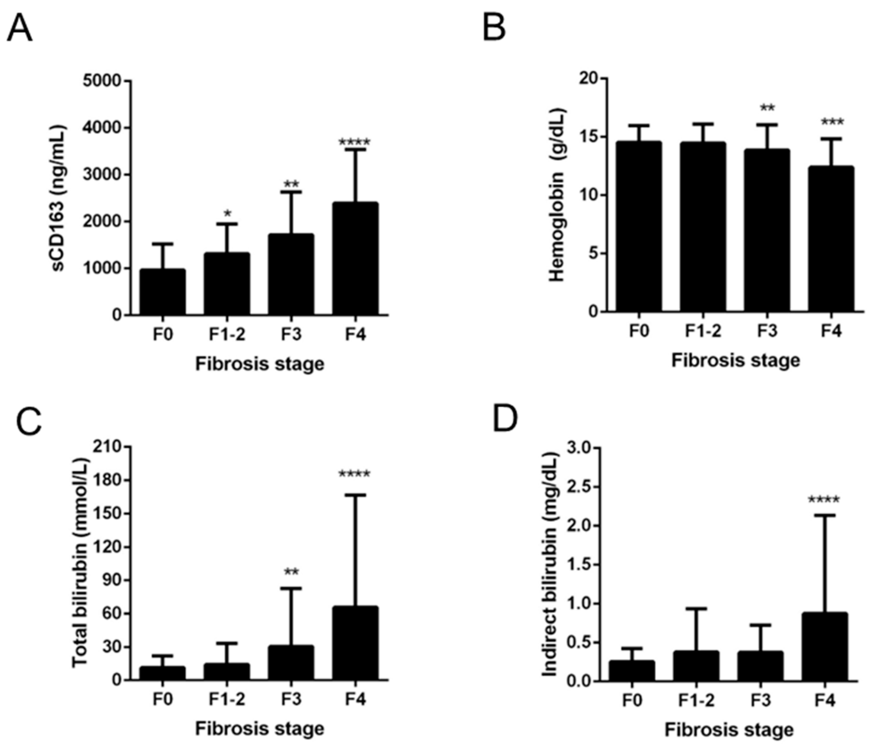

Suppl. Figure S4. Hemolysis and erythrophagocytosis increase with advancing liver fibrosis

Serum markers of erythrocyte turnover and macrophage-mediated erythrophagocytosis were analyzed according to histological fibrosis stage in 228 heavy drinkers. Shown are (A) soluble CD163, (B) hemoglobin, (C) total bilirubin, and (D) indirect bilirubin. Soluble CD163 increased progressively across histological fibrosis stages, from F0 (no fibrosis) to F4 (cirrhosis), indicating increasing hemoglobin–haptoglobin clearance and macrophage activation. In parallel, hemoglobin concentrations declined, whereas total and indirect bilirubin increased, particularly in advanced fibrosis and cirrhosis. The combined rise in sCD163 and indirect bilirubin together with falling hemoglobin supports the concept that disturbed erythrocyte turnover is an integral component of advanced chronic liver disease and the proposed RBC–liver axis. Data are shown as mean and 95% confidence interval. Asterisks indicate statistical significance versus F0 after adjustment for multiple comparisons using Dunnett's method: *$P < 0.05$, **$P < 0.01$, ***$P < 0.001$, ****$P < 0.0001$. Modified from [3]. F1 denotes portal fibrosis without septa, F2 portal fibrosis with few septa, and F3 numerous septa without cirrhosis.
